# Salivary Biomarkers for the Diagnosis of Sjögren’s Syndrome: A Review of the Last Decade

**DOI:** 10.3390/biomedicines13112664

**Published:** 2025-10-30

**Authors:** Virginia Ewa Lis, Anna Skutnik-Radziszewska, Ewa Zalewska, Anna Zalewska

**Affiliations:** 1 Department of Restorative Dentistry, Medical University of Bialystok, 15-089 Białystok, Poland; 2 Students’ Research Group Department of Restorative Dentistry, Medical University of Bialystok, 15-089 Białystok, Poland; 3 Independent Laboratory of Experimental Dentistry, Medical University of Bialystok, 15-089 Białystok, Poland

**Keywords:** Sjögren’s syndrome, saliva, salivary biomarkers, proteomics, metabolomics, autoantibodies, microRNA, extracellular vesicles, early diagnosis, autoimmune disease

## Abstract

**Objectives:** Sjögren’s syndrome (SjS) is a chronic autoimmune disease primarily affecting the salivary and lacrimal glands. Conventional diagnosis depends on invasive procedures, underscoring the need for non-invasive biomarkers. This systematic review summarizes evidence from 2014 to 2024 on the diagnostic and monitoring potential of salivary biomarkers in SjS. **Methods:** A systematic search of PubMed, Scopus, and Web of Science was performed according to PRISMA guidelines. Eligible human studies investigating salivary biomarkers in SjS were included. Data extraction and quality assessment were conducted independently by two reviewers. The protocol was registered in the OSF Registries. **Results:** Thirty-one studies were analyzed, identifying diverse metabolomic, proteomic, and molecular biomarkers. Consistent findings included increased levels of lactate, alanine, taurine, NGAL, β_2_-microglobulin, annexin A2, and regulatory RNAs (let-7i-5p, miR-17-5p), along with H19 ICR hypomethylation. Several extracellular vesicle (EV)-derived biomarkers demonstrated improved diagnostic stability and specificity. **Conclusions:** Saliva represents a promising, non-invasive diagnostic medium for Sjögren’s syndrome. Integrating multi-omics approaches-particularly EV-based analyses may enhance early diagnosis and personalized monitoring. Large, multicenter studies using standardized protocols are needed to validate these findings.

## 1. Introduction

### 1.1. Definition

**Figure 1:**
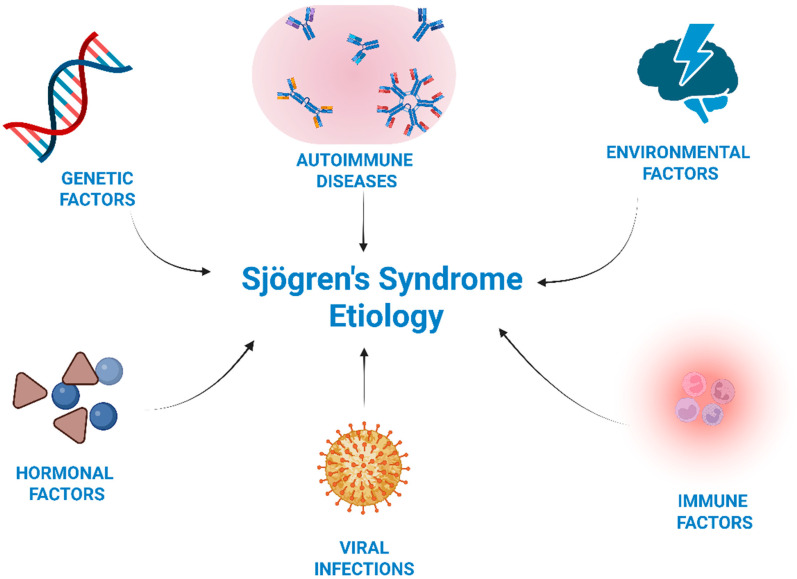
Etiopathogenesis of Sjögren’s syndrome. Schematic representation of the multifactorial etiology of Sjögren’s syndrome, showing the interaction between genetic predisposition, hormonal imbalance, immune dysregulation, and environmental triggers. Created in BioRender. Lis, V. (2025) https://BioRender.com/3w8ncqo.

Sjögren’s syndrome (SjS) is a chronic inflammatory autoimmune disease that manifests as a result of a combination of genetic, immunological, hormonal, and environmental factors (Figure 1) [1]. Genes correlated with the occurrence of SjS are associated with alterations in immune system function, particularly within innate immunity and inflammatory signaling pathways [2,3]. A strong correlation has been demonstrated with the presence of genes such as HLA Class II: alleles A1, B8, DRB103:01*, DQA105:01*, DQB102:01*, DRB115* (DR2), DR3, DQ2, DR5, and DR15/DQ1. These alleles reflect antigen presentation to CD4^+^ T cells, leading to excessive immune activation [4,5]. Studies show hormonal associations with Sjögren’s syndrome: lower basal DHEA-S, a higher cortisol/DHEA-S ratio, and reduced lifetime estrogen exposure, reflected by fewer cumulative menstrual cycles. Given the immunomodulatory role of DHEA-S, its deficiency may promote immune imbalance and chronic glandular inflammation. Diminished androgenic and estrogenic activity, combined with an altered cortisol-DHEA balance, may predispose to immune dysregulation and glandular hypofunction, thereby contributing to disease onset and progression [6,7]. It is also assumed that environmental factors, such as viral infections—particularly Epstein–Barr virus (EBV), cytomegalovirus (CMV), hepatitis C virus (HCV), and Coxsackievirus—in combination with genetic predispositions, may act as triggers for autoimmunity in primary Sjögren’s syndrome (pSS) by inducing chronic immune activation. This leads to increased levels of cytokines, such as type I interferons, B cell-activating factors, and chemokines, the elevation of which promotes the migration of lymphocytes and dendritic cells, resulting in chronic inflammation of the exocrine glands [8]. Antigen presentation occurs, activating autoreactive T and B cells. As a consequence, tissue damage ensues, further increasing exposure to autoantigens [9,10,11]. Stressful life events taking place before the onset of disease have been suggested as a potential risk factor for the development of autoimmune conditions such as Sjögren syndrome [12].

Sjögren’s syndrome manifests with changes affecting connective tissue. Inflammatory infiltrates develop, involving exocrine glands such as the lacrimal glands, salivary glands, sweat glands, mucous glands of the respiratory tract, vaginal glands, and gastrointestinal glands [13,14]. These infiltrates consist primarily of CD4^+^ T lymphocytes (helper Th1 and Th17), B lymphocytes, plasma cells, dendritic cells, and macrophages [15,16].

### 1.2. Symptoms

Infiltration of exocrine glands, particularly the lacrimal and salivary glands, leads to their gradual destruction. Glandular tissues gradually undergo transformation into fibrous connective tissue, resulting in impaired gland function (e.g., reduced tear or saliva production). This process is a key element in the pathogenesis of Sjögren’s syndrome [16,17,18,19,20].

Sjögren’s syndrome (SjS) can be defined as primary (pSjS) when it occurs independently. Secondary Sjögren’s syndrome (sSjS) when it develops in association with other systemic autoimmune diseases, such as rheumatoid arthritis, systemic lupus erythematosus, systemic sclerosis, primary biliary cirrhosis, autoimmune thyroid diseases, or multiple sclerosis [21,22,23].

**Figure 2:**
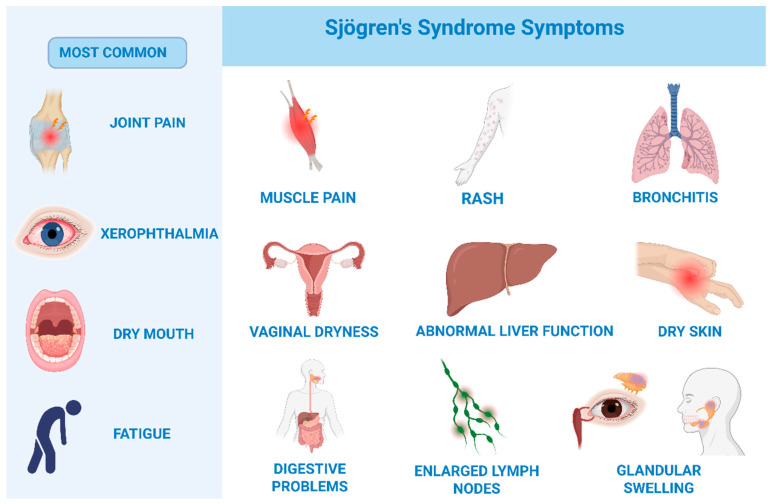
Clinical manifestations of Sjögren’s syndrome. Graphical overview summarizing the key clinical features of Sjögren’s syndrome. The image highlights both glandular symptoms, such as xerostomia and xerophthalmia, and extra-glandular manifestations including fatigue, arthralgia, pulmonary, hepatic, and neurological involvement. Created in BioRender. Lis, V. (2025) https://BioRender.com/g96c4zt.

The typical symptom triad includes dryness of the oral cavity (xerostomia) or eyes (xerophthalmia) (SICCA symptoms), fatigue, and joint pain. Patients may also present with additional systemic manifestations, such as vaginal dryness, dry skin, myalgia, interstitial lung disease, chronic bronchitis, bronchiolitis, pulmonary hypertension, lymphadenopathy, hematologic disorders (e.g., leukopenia, thrombocytopenia), chronic hepatitis, autoimmune hepatitis, primary biliary cirrhosis, nasal dryness, conjunctivitis, photophobia, disturbances in taste and smell, and photosensitivity [13,20] (Figure 2 [24]). The term SICCA refers to patients who present with symptoms of oral and ocular dryness (xerostomia and xerophthalmia) but do not meet the diagnostic criteria for primary Sjögren’s syndrome [25] [Table S1].

### 1.3. Epidemiology

A systematic literature review estimated the global incidence of Sjögren’s syndrome at 6.9 cases per 100,000 persons per year, with a prevalence of 60.8 diagnosed cases per 100,000 persons. SjS predominantly affects women, with an average female-to-male ratio ranging from 9:1 to 28:1. In women, the age at first diagnosis of pSjS typically falls between 55 and 65 years, whereas in men it is most often diagnosed after the age of 65 [18,26]. It is noteworthy that the first symptoms may appear many years before the diagnosis [17,18,20,27].

### 1.4. Rationale for the Review

Although knowledge of the mechanisms underlying Sjögren’s syndrome continues to expand, its diagnosis still depends largely on invasive procedures. These include minor salivary gland biopsy and serological testing for autoantibodies, which may be time-consuming, burdensome for patients, and often insufficient to detect the disease in its early or seronegative stages. Such limitations emphasize the urgent need for new, non-invasive, and clinically practical diagnostic tools. Saliva represents a particularly promising candidate, as it is easily obtainable and contains a wide range of molecules reflecting both local and systemic pathological processes. This systematic review aims to synthesize evidence from the past decade concerning salivary biomarkers in Sjögren’s syndrome, with a focus on their potential applications in diagnosis, disease monitoring, and the development of personalized therapeutic strategies.

## 2. Materials and Methods

### 2.1. Search Strategy and Data Extraction

The literature review was conducted for the period from 2014 to November 2024. The initial search was performed on 9 October 2024, and the final search on 25 November 2024.

The search strategy was conducted in accordance with the PRISMA (Preferred Reporting Items for Systematic Reviews and Meta-Analyses) guidelines [28]. The literature review included searches of the PubMed, Scopus, and Web of Science databases.

As part of the manual search, various combinations of keywords such as “Sjögren’s syndrome”, “saliva”, and “biomarkers” were used. To increase the sensitivity of the search strategy, suggested articles were also reviewed. Inclusion criteria comprised publications in English in peer-reviewed scientific journals that met the PECOS model (Population, Exposure, Comparison, Outcomes, Study Design) requirements (Table 1).

**Figure 3:**
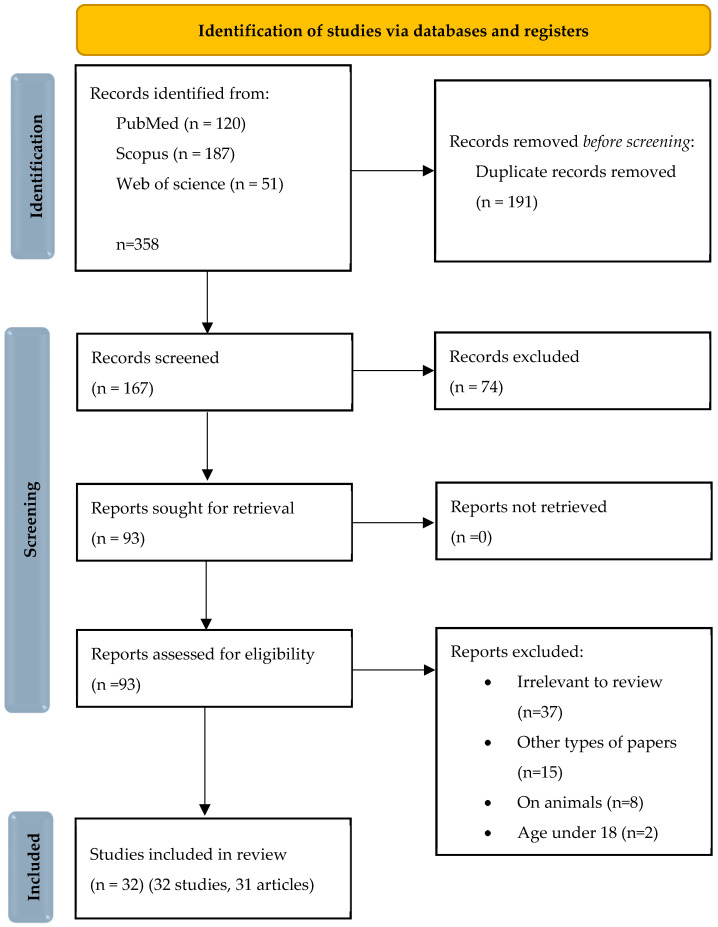
Flow diagram depicting the study selection process according to the PRISMA 2020 guidelines, including identification, screening, eligibility assessment, and inclusion of studies in the review. PRISMA 2020 flow diagram https://www.prisma-statement.org/prisma-2020-flow-diagram (accessed on 29 October 2025) [29]. This work is licensed under CC BY 4.0. To view a copy of this license, visit https://creativecommons.org/licenses/by/4.0/.

Records were analyzed by two independent researchers (V.L. and A.Z.), minimizing the risk of selection bias. They were first assessed based on titles and abstracts, and subsequently, if they met the inclusion criteria, the full texts were reviewed. Articles deemed irrelevant after abstract or full-text analysis were excluded. A detailed search flow diagram is presented in the PRISMA 2020 flow diagram in Figure 3.

### 2.2. Registration

This Review Was Prospectively Registered in the OSF REGISTRIES (Open Science Framework) Under the Identifier FZX4T (https://doi.org/10.17605/OSF.IO/FZX4T) [30].

The full review protocol, including the eligibility criteria, search strategy, and data extraction plan, is openly accessible at the registration website. No amendments to the protocol were introduced after registration. Minor editorial clarifications were made, which did not affect the predefined objectives, inclusion/exclusion criteria, or analysis strategy.

### 2.3. Quality Assessment and Critical Appraisal for the Systematic Review of Included Studies

The risk of bias in individual studies was assessed using quality assessment tools developed by the National Heart, Lung, and Blood Institute, part of the National Institutes of Health [31]. Two independent researchers (V.L. and A.Z.) completed the risk of bias assessment forms. The quality assessment results for each study are presented in Figure S1. The most frequently identified issues included blinded participant status and sample size justification, which were absent in all studies. No randomization was observed in 27 studies.

The level of evidence was determined according to the classification developed by the Oxford Centre for Evidence-Based Medicine, focusing on diagnosis [32]. The critical appraisal was performed by assigning points for each risk criterion: 1 for low risk, 0.5 for unclear risk, and 0 for high risk.

Of the 32 studies analyzed (31 articles, with one article including two studies), all were deemed to be of “good” quality. All studies were classified as level three or four in the five-level hierarchy of evidence.

## 3. Results

A total of 31 studies published between 2014 and 2024 were included in this systematic review. These investigations collectively explored a wide spectrum of salivary biomarkers associated with Sjögren’s syndrome (SjS), employing diverse analytical methods such as mass spectrometry, NMR spectroscopy, ELISA, qPCR, and next-generation sequencing. The classification criteria, diagnostic scores, and detailed study characteristics are summarized in Supplementary Materials (Table S2).

To provide a clearer and more integrated presentation, the results are grouped according to the main biomarker categories summarized in the tables: (1) Metabolomics Analysis, (2) Salivary Proteomics, (3) Molecular Biomarkers (microRNAs, RNA transcripts, DNA methylation), (4) Autoimmune Biomarker Panel, and (5) Enzymatic Markers in Saliva.

### 3.1. Metabolomic

#### 3.1.1. Lactate, Alanine, and Malate

In the study by Piacenza Florezi et al. [33] a significant increase in the concentrations of metabolites such as lactate, alanine, and malate was demonstrated. These metabolites are products of bioenergetic pathways related to anaerobic glycolysis and oxidative stress [34,35].

The high salivary lactate levels observed in patients with Sjögren’s syndrome reflect chronic hypoxia and inflammation within the salivary glands, leading to enhanced glycolysis and the accumulation of acidic metabolites. These alterations promote epithelial cell apoptosis and intensify the local recruitment of macrophages and lymphocytes, which constitutes a pathogenic mechanism sustaining the inflammatory process [36].

#### 3.1.2. Leucine, Valine, and Isoleucine

The elevated levels of branched-chain amino acids—leucine, valine, and isoleucine—as well as arginine may be associated with the activation and proliferation of T and B lymphocytes within salivary gland infiltrates. Activated lymphocytes exhibit an increased demand for amino acid metabolism, which is essential for energy production and the synthesis of regulatory proteins involved in immune responses. These alterations therefore reflect the bioenergetic profile characteristic of chronic autoimmunity in Sjögren’s syndrome [33].

#### 3.1.3. Glucose, Glycerol, and Taurine

In the study by Alt-Holland et al. [37], it was demonstrated that patients with Sjögren’s syndrome exhibit significantly increased concentrations of glucose, glycerol, and taurine, accompanied by reduced levels of short-chain fatty acids. The elevation in glucose levels may result from impaired transport and metabolism within the salivary gland epithelium as well as dysfunction of the salivary barrier. Taurine, an abundant free amino acid in humans, performs multiple biological functions, including antioxidant activity, osmoregulation, membrane stabilization, maintenance of calcium concentrations, and cellular homeostasis. High taurine concentrations are typically detected in tissues subjected to chronic inflammation and oxidative stress. Therefore, taurine may serve a compensatory role in mechanisms underlying the loss of secretory function in patients with Sjögren’s syndrome [38].

#### 3.1.4. Metabolomic

In turn, studies by Bosman et al. [39] and Setti et al. [40] indicated that the salivary metabolite profile in Sjögren’s syndrome includes alterations in amino acid metabolism pathways and the citric acid cycle. Within the citric acid cycle, two carboxylic acids exhibit decreased expression in SS. The malic acid, which is synthesized through the citric acid cycle, and succinic acid, an important component of this cycle and therefore essential for the production of adenosine triphosphate [39]. Significantly elevated levels of alanine, isovaleric acid, and succinic acid were observed, demonstrating high sensitivity and specificity for disease diagnosis. The fact that numerous studies consistently report increased concentrations of alanine, lactate, and taurine further confirms that oxidative stress processes and energy metabolism disturbances in epithelial cells are key elements in the pathogenesis of Sjögren’s syndrome [34,35,41].

#### 3.1.5. Tryptophan, Tyrosine, and Aspartate

Salivary metabolomic analysis conducted by Li et al. [42] revealed significant alterations in tryptophan, tyrosine, and aspartate metabolism. Aspartic acid, an excitatory amino acid, exerts a potent activating effect on neurons. Increased concentrations of aspartic acid and phenylalanine in the brain have been associated with behavioral and cognitive impairments. In pSjS, distal sensory and sensorimotor neuropathies are the most frequent forms of peripheral nerve involvement, and their findings suggest that elevated aspartic acid levels may contribute to these neurological manifestations [42]. Tryptophan and tyrosine metabolites are thought to influence the immune-neuroendocrine axis, and their dysregulation may be associated with neurological symptoms and fatigue in patients with SjS [42].

#### 3.1.6. Salivary Adiponectin

The study by Tvarijonaviciute et al. [43] demonstrated that adiponectin is significantly elevated in the saliva of patients with Sjögren’s syndrome. Adiponectin is a protein belonging to the soluble defence collagen superfamily. It is mainly synthesized and secreted by adipose tissues and, to a lesser extent, by non-adipocyte cells, including salivary gland cells [44]. Adiponectin exerts anti-inflammatory effects and may limit apoptosis of glandular cells. The potentially serving as a compensatory mechanism to counteract tissue damage. The positive correlation between adiponectin levels and the severity of oral dryness symptoms supports its association with disease pathology.

### 3.2. Salivary Proteomics

#### 3.2.1. Neutrophil Gelatinase-Associated Lipocalin (NGAL)

One of the important findings is the elevation of neutrophil gelatinase-associated lipocalin (NGAL) both in the saliva and in the epithelial cells of salivary glands in patients with primary Sjögren’s syndrome (pSS) [45]. NGAL plays a key role in innate immunity-it binds iron, inhibits bacterial growth, activates neutrophils, and modulates the production of proinflammatory cytokines. Increased NGAL levels within the glands were positively associated with the severity of inflammatory infiltrates and the presence of adipose tissue in the salivary gland parenchyma. These observation suggest that this protein may contribute to the progression of inflammation and destruction of glandular tissue [46].

#### 3.2.2. S100 Protein Family, Annexin A2, and CD14

Studies by Finamore et al. [47] demonstrated that extracellular vesicles (EVs) from Sjogren syndrome patients contain a range of proteins associated with inflammatory responses and the activation of innate immune cells, including S100A11, S100A12, annexin A2, and CD14. S100 proteins are involved in modulating neutrophil and macrophage responses and in activating RAGE and TLR4 receptors, which leads to the secretion of proinflammatory cytokines (e.g., IL-6, TNF-α) [48]. Annexin A2, in turn, regulates phagocytosis and antigen presentation processes, thereby promoting the persistence of chronic inflammation. Elevated levels of CD14 reflect the activation of monocytes and macrophages, which play a key role in initiating the autoimmune response.

#### 3.2.3. MUC5B, Prolactin-Inducible Protein (PIP)

In the study by Di Giorgi et al., it was confirmed that in the saliva of patients with primary Sjögren’s syndrome (pSjS), as well as in individuals in the preclinical phase of the disease (positive for SSA/Ro antibodies but without clinical symptoms), there are alterations in the expression of MUC5B, prolactin-inducible protein (PIP), cystatin C, and lipocalin-1 [49]. MUC5B is a mucin responsible for the rheological properties of saliva. Its overexpression indicates epithelial remodeling and secretory dysfunction [50]. PIP is involved in regulating immune responses and mediating interactions between epithelial cells and lymphocytes, and its altered levels reflect autoimmune processes [49]. The presence of these changes in the preclinical phase suggests that proteomic disturbances emerge at a very early stage of the disease, before clinical symptoms become apparent.

#### 3.2.4. β2-Microglobulin

β2-microglobulin is a component of MHC class I molecules, and its increase indicates activation of antigen presentation and chronic infiltration of T and B cells in the salivary glands. It was demonstrated that salivary β2-microglobulin positively correlates with the ESSPRI index, which reflects the severity of clinical symptoms of the disease [51,52]. This correlation suggests that β2-microglobulin may serve as a marker of disease activity and the progression of tissue damage.

#### 3.2.5. Thrombospondin 1

The studies by Delaleu et al. [49,53] demonstrated that patients with ectopic germinal centers (GCs) exhibit a distinct salivary proteomic profile. Elevated concentrations of thrombospondin-1 are characteristic of this group. Thrombospondin-1 participates in cell adhesion, regulation of angiogenesis, and the creation of a microenvironment conducive to B-cell activation [54]. Ectopic germinal centers increase the risk of B-cell transformation and the development of non-Hodgkin lymphoma, which is why the presence of these biomarkers also has important prognostic significance [55].

### 3.3. Molecular Biomarkers

#### 3.3.1. Non-Coding RNA

Studies conducted by Cross et al. [56] provide evidence of the richness of non-coding RNAs present in extracellular vesicles (EV) in saliva of patients with Sjögren’s syndrome. In total, more than 1475 transcripts were identified that differed between the patient group and healthy controls, nearly half of which were ncRNAs, including numerous lncRNAs, tRNAs, and miRNAs. Notably, the more than two-fold reduction in tRNA-Ile-AAT-2-1 levels compared to the control group represents a promising premise for further investigation of this tRNA as a disease biomarker.

Alterations in the content of tRNAs and other small RNA molecules transported by extracellular vesicles (EVs) may reflect disruptions in the homeostasis of salivary gland epithelial cells. Importantly, extracellular vesicles protect RNA from degradation, making them an exceptionally stable material for diagnostic purposes [57].

#### 3.3.2. Imprinting Control Region (ICR) of the H19 Locus

The study by Karagianni et al. [58] shows that epigenetic changes play an important role in Sjögren’s syndrome, especially DNA methylation. The authors found reduced methylation in the imprinting control region (ICR) of the H19 gene in DNA taken from the saliva of patients compared to healthy controls. H19 encodes a long non-coding RNA (lncRNA) that helps regulate the expression of genes involved in inflammation. LncRNA H19 is an important factor in immune system imbalance. It contributes to the development of autoimmune diseases and their symptoms. It regulates the production of pro-inflammatory and anti-inflammatory cytokines by interacting with key immune pathways, such as the NF-κB signaling pathway and different microRNA networks. In addition, lncRNA H19 supports immune tolerance by influencing T cell differentiation and maintaining the balance between regulatory T cells (T-regs) and T helper 17 (Th17) cells [59,60]. Hypomethylation of the ICR leads to higher H19 transcription, which in autoimmune diseases may increase lymphocyte activation and worsen damage to the glandular epithelium [61].

#### 3.3.3. microRNA

Studies by Sembler-Møller et al. [62] demonstrated that patients with primary Sjögren’s syndrome have a distinct salivary microRNA expression profile, clearly different from individuals experiencing oral dryness symptoms (SICCA). Notably, the combination of reduced miR-17-5p levels and increased let-7i-5p expression showed excellent diagnostic performance, underscoring the potential of these molecules as specific differential biomarkers [63]. Let-7i-5p is a member of the Lethal-7 microRNA family. It exerts regulatory effects via direct targeting of genes implicated in collagen metabolism, TGF-beta signaling pathways, DNA repair mechanisms, cellular proliferation and differentiation, ubiquitination processes, gene silencing, and maintenance of oxygen homeostasis [64]. In their 2020 study, Najm et al. [65] provided evidence that miR-17 exerts anti-inflammatory and anti-erosive effects in vivo. These properties are mediated by the inhibition of the IL-6 family autocrine amplification loop, achieved through direct targeting of JAK1 and STAT3.

### 3.4. Autoimmune Biomarker Panel

#### 3.4.1. Monomeric and Polymeric Anti-SSA/Ro52 Immunoglobulin A1 Isoforms

Another particularly noteworthy aspect is the differentiation of immunoglobulin A1 isoforms. In a study conducted by Chiang et al. [66], the presence of monomeric and polymeric IgA1 isoforms targeting SSA/Ro52 was assessed in the saliva of patients with primary Sjögren’s syndrome (pSjS) and individuals exhibiting SICCA symptoms without fulfilling the diagnostic criteria for the disease. Among patients with pSjS, polymeric IgA1 (pIgA1) predominated, instead monomeric IgA1 (mIgA1) was more prevalent in SICCA subjects. The presence of polymeric IgA1 showed a significant correlation with the degree of lymphocytic infiltration observed in salivary gland biopsy specimens. Polymeric IgA1 is associated with the activation of FcαRI receptors and the induction of proinflammatory signaling within the salivary glands. In contrast, monomeric IgA1 appears to exert mainly regulatory and anti-inflammatory functions [67]. This distinct isoform profile suggests that analyzing their relative proportions in saliva may represent a novel approach for differentiating pSjS from other causes of mucosal dryness and for evaluating disease activity.

#### 3.4.2. Inducible T Cell Co-Stimulator (ICOS)

The inducible T-cell co-stimulator (ICOS) is a co-stimulatory molecule belonging to the CD28 family, playing a role in T-cell activation and humoral immune responses. ICOS is particularly critical for the differentiation of T follicular helper (Tfh) cells. Tfh cells, activated via ICOS signaling, promote the maturation and proliferation of B lymphocytes, thereby contributing to excessive autoantibody production and the development of lymphocytic infiltrates. Consequently, ICOS may be considered as an important pathogenic factor in primary Sjögren’s syndrome [68].

Li et al. [69] investigated ICOS concentrations both in serum and saliva of patients with primary Sjögren’s syndrome. They observed that ICOS levels were significantly elevated in affected individuals compared to healthy controls, and that higher ICOS concentrations were inversely correlated with salivary volume.

#### 3.4.3. Interleukin 6

IL-6 plays a role in the pathogenesis of Sjögren’s syndrome. It promotes the differentiation of B lymphocytes into plasma cells that produce autoantibodies, activates T lymphocytes, and induces the expression of adhesion molecules and chemokines. Prolonged exposure to high IL-6 concentrations leads to sustained chronic inflammation of the salivary glands, fibrosis, and irreversible loss of their secretory function. Therefore, measuring IL-6 in saliva may reflect local inflammatory activity and the extent of tissue damage [70]. Moreno-Quispe et al. [71] demonstrated that in patients with pSS, salivary IL-6 concentrations were significantly higher than in healthy individuals, indicating enhanced local production of this cytokine within the salivary glands. Elevated IL-6 levels reflect the activation of T- and B-cell axes. IL-6 acts synergistically with other cytokines, including IL-21 and type I interferons, supporting B-cell maturation and the production of anti-SSA and anti-SSB antibodies. Consequently, IL-6 concentrations may correlate with autoantibody titers and the severity of clinical symptoms. Moreover, IL-6 increases the expression of adhesion molecules and chemokines, facilitating the migration of effector cells into the salivary glands and contributing to the development of chronic inflammation [70]. In summary, elevated salivary IL-6 levels in patients with Sjögren’s syndrome reflect active local inflammation of the salivary glands, promote B-cell maturation and autoantibody production, and are associated with greater clinical severity [72,73,74]. Assessing this parameter may be useful in diagnosis, monitoring disease activity, and guiding therapeutic strategies.

#### 3.4.4. Tissue-Specific Autoantibodies

Conventional biomarkers like anti-SSA and anti-SSB antibodies are widely used but have limitations. The presence of them typically indicates advanced disease and systemic involvement [75]. Relying on them alone can miss early or seronegative cases. Studies show that TSAs were detected even earlier than anti-SSA and anti-SSB antibodies [76].

Tissue-specific autoantibodies (TSAs), such as anti-CA6, anti-SP1, and anti-PSP, are promising markers because they target antigens mainly found in the salivary glands, where pSS-related damage typically begins. Jin et al. [77] demonstrated that elevated concentrations of these autoantibodies were detected both in the serum and saliva of patients with pSS, and their presence was particularly diagnostically relevant in patients who were seronegative for the classical anti-SSA and anti-SSB autoantibodies. Their studies suggest that the presence of these antibodies reflects local salivary gland damage and may represent a valuable diagnostic tool in the early stages of the disease. Moreover, they showed that the concentrations of these autoantibodies vary depending on disease duration with a negative time correlation. These antibodies are directed against proteins present in the epithelial cells of the salivary glands, and their detection reflects localized tissue injury [78]. From a clinical perspective, tissue-specific autoantibodies (TSAs) may serve as an additional marker supporting the diagnosis of Sjögren’s syndrome, particularly in early and seronegative cases.

#### 3.4.5. Soluble Siglec-5

Siglec-5 belongs to the family of sialic acid-binding immunoglobulin-like lectins, which are receptors involved in regulating inflammatory responses and inhibiting excessive immune system activation. Under physiological conditions, siglec-5 serves an immunoregulatory role by limiting the activation of neutrophils and monocytes [79]. Elevated siglec-5 levels may indicate chronic activation of the innate immune response along with a compensatory attempt to restrain inflammation. Lee et al. [80] observed that siglec-5 concentrations were significantly elevated in patients with primary Sjögren’s syndrome (pSS) compared to healthy individuals and patients with other autoimmune diseases. In clinical practice, the measurement of siglec-5 in saliva may support differential diagnosis and monitoring of disease activity, as it is positively associated with reduced saliva production and damage to the ocular surface [79].

#### 3.4.6. Free Light Chains (FLCs)

Free immunoglobulin light chains (FLCs) are byproducts of intense B-cell activation and immunoglobulin synthesis. Under physiological conditions, small amounts of FLCs are present in plasma and other body fluids, and their concentrations increase during enhanced humoral immune responses or clonal proliferation of plasma cells. In the context of primary Sjögren’s syndrome (pSjS), their presence in saliva is of particular relevance, as it reflects pathological processes occurring locally within the secretory glands. A study by Sandhya et al. [81] demonstrated that both kappa and lambda FLC levels were significantly elevated in the saliva of patients with pSjS compared to healthy individuals. Within the salivary glands, ectopic structures resembling B-cell germinal centers—so-called ectopic germinal centers—are formed. In these areas, B cells become activated and differentiate into plasma cells, leading to local overproduction of immunoglobulins. During this process, excess kappa and lambda light chains that are not incorporated into complete immunoglobulin molecules are released into the tissue environment and subsequently enter the saliva. Since saliva reflects the local milieu of the glands, the measurement of FLC may be more specific for pSS than serum parameters, which are influenced by other sources of immunoglobulins [82,83].

### 3.5. Enzymatic Markers in Saliva

#### 3.5.1. Dipeptidyl Peptidase-4 (DPP4/CD26)

Studies conducted by Garreto and colleagues [84] demonstrated that in the saliva of patients suffering from Sjögren’s syndrome, there is a significant increase in both the activity and concentration of dipeptidyl peptidase-4 (DPP4/CD26). DPP4 is a serine protease, also known as the complement differentiation protein (CD26). This enzyme is expressed on the surface of most mammalian cells, as well as bacteria, fungi, and plants [61]. In the immune system, DPP4 is mainly present on the surface of T lymphocytes, NK cells, B lymphocytes, and myeloid lineage cells. The highest levels of expression are observed in Th17 T lymphocytes and in T cells associated with mucosa-associated lymphoid tissue (MALT). NK cells initially exhibit low surface expression of DPP4; however, its levels increase upon stimulation with interleukins [85]. In addition to its proteolytic activity, DPP4/CD26 also functions as a cell surface receptor, participates in intracellular signal transduction, mediates cell adhesion, and acts as a costimulatory protein involved in the development, stimulation, and activation of T lymphocytes. High CD26 expression is associated with the differentiation of Th1 and Th17 lymphocytes [86]. Its excessive presence may contribute to the persistence of chronic inflammation within the salivary glands and to enhanced lymphocytic infiltration of exocrine glands, which constitutes a key mechanism in the pathogenesis of Sjögren’s syndrome. Elevated concentrations of dipeptidyl peptidase-4/CD26 have been observed in the serum of individuals with type 1 diabetes, rheumatoid arthritis, systemic lupus erythematosus, and inflammatory bowel diseases, which suggests its involvement in the pathogenesis of autoimmune disorders [87]. In view 3of these observations, targeting DPP4 as a salivary biomarker in patients with Sjögren’s syndrome holds significant diagnostic and therapeutic potential.

#### 3.5.2. Matrix Metalloproteinase-9 (MMP9), Neutrophil Elastase (ELANE), Cathepsin G (CTSG), and Myeloblastin (PRTN3)

Garreto and colleagues [84] also revealed a significant increase in the levels of Matrix Metalloproteinase-9 (MMP9), Neutrophil Elastase (ELANE), Cathepsin G (CTSG), and Myeloblastin (PRTN3) in the saliva of patients with Sjögren’s syndrome compared to healthy controls. Matrix metalloproteinases (MMPs), including MMP9, are responsible for the degradation of structural proteins within the extracellular matrix, such as collagen and elastin. This process contributes both to the remodeling and destruction of glandular tissue. At the same time, matrix degradation products exert chemotactic effects on inflammatory cells, which further sustains the inflammatory response. Therefore, increased activity of MMP9 and other proteases reflects both the damage to the salivary gland structure and the active inflammatory processes characteristic of Sjögren’s syndrome [88].

The identification of elevated levels of proteases such as neutrophil elastase, cathepsin G, myeloblastin, and trypsin exclusively in saliva samples from Sjögren’s syndrome patients indicates that the mechanisms of neutrophil and immune cell degranulation play a significant role in the disease [89,90].

These enzymes not only degrade the extracellular matrix but also modify epithelial cell surface receptors, facilitating their apoptosis and the exposure of antigens, which intensifies autoimmune processes. States like this promotes the induction of an autoimmune response against the body’s own glandular structures [91].

#### 3.5.3. α-Enolase (ENO1)

The study by Wei et al. [92] demonstrated a significant increase in the expression of α-enolase (ENO1) in the saliva of patients with Sjögren’s syndrome. ENO1 is a glycolytic pathway enzyme that plays an important role in cellular energy metabolism. It also has a key function in various biological and pathological processes associated with both cancer and autoimmune diseases [93].

It functions as an autoantigen against which ENO1 autoantibodies are produced [94]. Elevated levels of anti-ENO1 autoantibodies correlate with a decreased salivary secretion rate, suggesting that the autoimmune response directed against this protein may contribute to damage of the secretory epithelium and the development of hyposalivation-one of the primary symptoms of Sjögren’s syndrome. However, the precise role of ENO1 in the pathogenesis of pSS warrants further study.

## 4. Discussion

### 4.1. Metabolomic

Metabolomic studies have consistently shown marked disruptions in amino acid and energy metabolism in the saliva of patients with Sjögren’s syndrome. Increased levels of lactate, taurine, and alanine point to mitochondrial dysfunction and oxidative stress in the salivary glands, while reduced amounts of choline and acetate may indicate altered phospholipid metabolism and imbalances in the oral microbiome. Together, these findings suggest a systemic redox imbalance that may underlie glandular hypofunction [34,35]. Most available studies were carried out on small patient groups and used different analytical approaches, which makes their results difficult to compare. Future research should focus on applying standardized methods for sample preparation and validation to establish consistent metabolomic profiles that could support early diagnosis of the disease.

### 4.2. Salivary Proteomics

Proteomic studies consistently demonstrate increased expression of proteins related to immune response and inflammation, including β_2_-microglobulin, S100A8/A9, NGAL, and annexin A2 [47,51,52]. These findings highlight the crucial role of epithelial inflammation and innate immune activation in the development of Sjögren’s syndrome. Interestingly, studies focusing on proteomes derived from extracellular vesicles (EVs) have shown higher diagnostic accuracy than whole-saliva analyses, suggesting that EVs represent a promising and stable source of biomarkers [47].

However, differences in protein identification techniques and the lack of standardized normalization across proteomic workflows remain significant challenges. Integrating EV proteomic data with clinical parameters and disease activity indices may help translate these biomarkers into practical diagnostic tools.

#### Exosome-like Vesicles EVs

Until recently, research primarily focused on analyzing the composition of non-stimulated (NWS) and stimulated (SWS) whole saliva. However, in 2008, Ogawa et al. [95] published an article demonstrating that human saliva contains exosome-like vesicles (EVs). Studies on the composition of EVs have provided evidence that they may be valuable in the diagnosis and analysis of saliva in patients. Multiple investigations [96,97] have confirmed that the chemical composition of EVs differs from that of NWS and SWS, thereby opening new avenues for expanding diagnostic capabilities. EVs represent a heterogeneous group of membrane-associated nanoparticles released by cells into the extracellular space. Their primary components include lipids, proteins, and nucleic acids, whose profiles reflect both the cellular origin and the mechanisms of EV biogenesis [56]. Isolation of extracellular vesicles (EVs) enables the acquisition of an exceptionally precise and stable protein profile associated with inflammation and immune response, thereby significantly enhancing the reliability of analyses. The integration of conventional salivary proteomics with EV studies and advanced analytical techniques opens new perspectives for understanding the pathomechanisms of primary Sjögren’s syndrome, facilitates the identification of novel breakthrough biomarkers, and allows for more effective monitoring of disease progression and therapeutic response [98].

### 4.3. Molecular Biomarkers

Growing molecular evidence indicates that gene regulation and epigenetic mechanisms play a significant role in the pathogenesis of Sjögren’s syndrome. Dysregulated expression of microRNAs (such as let-7i-5p, miR-17-5p, miR-23a, and miR-181a) and hypomethylation of immune-related regions, including H19 ICR, point to impaired immune tolerance and abnormal lymphocyte activation [56]. These molecular alterations may represent early and non-invasive indicators of disease development.

However, the majority of current studies are based on small discovery cohorts and lack external validation. To better understand the regulatory networks underlying glandular dysfunction, future research should employ longitudinal, multi-omics approaches that integrate transcriptomic, epigenetic, and proteomic data.

### 4.4. Autoimmune Biomarker Panel

Salivary detection of classic autoantibodies, including anti-SSA/Ro and anti-SSB/La, remains of diagnostic importance; however, emerging evidence indicates that certain salivary immunoglobulin isoforms, such as IgA1 anti-Ro52, may offer greater sensitivity in mucosal samples [56].

Despite these promising findings, significant variability in assay methodologies, analytical platforms, and cutoff thresholds continues to hinder their clinical standardization and widespread implementation. Therefore, future studies should perform parallel comparisons of serum and salivary antibody profiles to accurately determine the diagnostic value and reliability of salivary autoantibody panels in Sjögren’s syndrome.

### 4.5. Enzymatic Markers in Saliva

Several enzymes associated with tissue remodeling and immune regulation- including amylase, adenosine deaminase, and dipeptidyl peptidase-4—have been proposed as potential salivary biomarkers of glandular dysfunction [89,90,92]. Their increased activity likely reflects epithelial injury and compensatory secretory responses that occur during chronic inflammation.

However, these enzymatic markers lack specificity and can be affected by concomitant systemic or oral conditions. Therefore, large, well-controlled studies incorporating both healthy and sicca participants are needed to validate their diagnostic utility and define standardized reference ranges for clinical use.

### 4.6. Integrative Perspective and Limitations

Overall, the available evidence demonstrates that salivary analyses offer valuable information on both local and systemic mechanisms involved in the pathogenesis of Sjögren’s syndrome. Consistent findings across metabolomic, proteomic, and molecular investigations emphasize the potential of a multi-omics approach to better delineate disease heterogeneity and underlying biological pathways. Nevertheless, most studies to date remain exploratory and cross-sectional, with considerable methodological variation and limited reproducibility. A key limitation is the lack of harmonized protocols for saliva collection, processing, and biomarker quantification.

Recent data suggest that extracellular vesicles (EVs)-based analyses may help to overcome these limitations by providing stable and cell-specific molecular profiles. Further standardization and validation of these integrative approaches are required to ensure reproducible results and to translate salivary biomarkers into clinically applicable diagnostic tools.

## 5. Conclusions

Saliva is increasingly recognized as a valuable, non-invasive diagnostic biofluid that reflects the inflammatory, metabolic, and immune alterations characteristic of Sjögren’s syndrome. Recent advances in metabolomic, proteomic, and molecular profiling have enabled the identification of multiple promising salivary biomarkers. Among them, metabolites, proteins, and regulatory RNAs—such as ICOS transcripts, DNA methylation signatures, and microRNAs (e.g., let-7i-5p, miR-17-5p)-show particular diagnostic potential.

However, the current body of evidence remains constrained by small cohorts and methodological variability. Future studies should prioritize large-scale, multicenter, and longitudinal designs employing standardized analytical protocols and integrating biomarker dynamics with clinical disease activity measures. Particular attention should be given to extracellular vesicle (EV)-based analyses, as their stable and cell-specific molecular content offers improved diagnostic reliability and the opportunity to identify novel biomarkers for early and personalized detection of Sjögren’s syndrome.

## Figures and Tables

**Table 1:** Inclusion and exclusion criteria applied in the systematic review. Summary of eligibility parameters used for study selection according to the PECOS framework (Population, Exposure, Comparison, Outcomes, Study Design).

Parameter	Inclusion Criteria	Exclusion Criteria
Population	Results of studies on humans; patients age from 18 to 99 years, both genders	Results of studies without human participants (e.g., studies on animals or in vitro)
Exposure	Sjögren’s syndrome	Only SICCA
Comparison	Not applicable	
Outcomes	Saliva components as potential biomarkers	Salivary gland biopsy
Study design	Original research articles, pilot studies, and letters to the editor published in English since 2014	Literature reviews, case reports, commentaries and others in a language other than English

## Data Availability

No new data were created or analyzed in this study. Data sharing is not applicable to this article. All data supporting the findings of this review are available in the cited publications included in the systematic analysis.

## Supplementary Materials

The following supporting information can be downloaded from https://www.mdpi.com/article/10.3390/biomedicines13112664/s1, Figure S1: Quality assessment of the included studies and the main potential sources of bias.; Table S1: Diagnostic criteria of Sjogren syndrome; Table S2: Results [96,97,98,99,100,101,102,103,104,105,106,107,108,109].
