# Supplementary material for: Salivary Biomarkers for the Diagnosis of Sjögren’s Syndrome: A Review of the Last Decade
Source: Biomedicines. 2025 Oct 30;13(11):2664. doi: 10.3390/biomedicines13112664 (PMC12649840; doi:10.3390/biomedicines13112664)
Supplement: Supplementary file 1 [file biomedicines-13-02664-s001.zip › Figure S1. Quality assessment, including the main potential risk of bias.pdf]

Figure S1. Quality assessment of the included studies and the main potential sources of bias.

Study	clearly stated research question or objective	clearly defined study population	sample size justification	groups recruitment from the same population	valid inclusion and exclusion criteria	cases differentiated from controls	randomization	clearly defined measures	blinded participant status	adjusted statistical methods	summary quality score
Piacenza Florezi et al. Brazil 2024 [1]	✓	✓	△	✓	✓	✓	✗	✓	✗	✓	✓
Vyas et al. USA 2024 [2]	✓	✓	△	✓	✓	✓	✗	✓	✗	✓	✓
Alt-Holland et al. USA 2023 [3]	✓	✓	△	✓	✓	✓	△	✓	✗	✓	✓
Alt-Holland et al. USA 2023 [3]	✓	✓	△	✓	✓	✓	△	✓	✗	✓	✓
Bosman et al. France, Brasil 2023 [4]	✓	✓	△	✓	✓	✓	✗	✓	✗	✓	✓
Setti et al. Italy 2023 [5]	✓	✓	△	✓	✓	✓	✗	✓	✗	✓	✓
Li et al. 2022 China [6]	✓	✓	△	✓	✓	✓	△	✓	✗	✓	✓
Herrala et al. Finland 2021 [7]	✓	✓	△	✓	✓	✓	✗	✓	✗	✓	✓
Tvarijonaviciute et al. 2019 Spain [8]	✓	✓	△	✓	✓	✓	✗	✓	✗	✓	✓
Kageyama et al. Japan 2015 [9]	✓	✓	△	✓	✓	✓	✗	✓	✗	✓	✓
Tian et al. China 2024 [10]	✓	✓	△	✓	✓	✓	△	✓	✗	✓	✓
Giorgi et al. 2022 Italy [11]	✓	✓	△	✓	✓	✓	✗	✓	✗	✓	✓
Finamore et al. 2021 Italy	✓	✓	△	✓	✓	✓	✗	✓	✗	✓	✓

Study	clearly stated research question or objective	clearly defined study population	sample size justification	groups recruitment from the same population	valid inclusion and exclusion criteria	cases differentiated from controls	randomization	clearly defined measures	blinded participant status	adjusted statistical methods	summary quality score
[12]											
Aqrawi et al. Norway 2020 [13]	✓	✓	△	✓	✓	✓	✗	✓	✗	✓	✓
Chen et al. Norway2019 [14]	✓	✓	△	✓	✓	✓	✗	✓	✗	✓	✓
Cecchettini et al. 2019 Italy [15]	✓	✓	△	✓	✓	✓	✗	✓	✗	✓	✓
Aqrawi et al. Norway 2019 [16]	✓	✓	△	✓	✓	✓	✗	✓	✗	✓	✓
Garza-García et al. Mexico 2017 [17]	✓	✓	△	✓	✓	✓	✗	✓	✗	✓	✓
Aqrawi et al. Norway 2017 [18]	✓	✓	△	✓	✓	✓	✗	✓	✗	✓	✓
Delaleu et al. Switzerland 2016 [19]	✓	✓	△	✓	✓	✓	✗	✓	✗	✓	✓
Deutsch et al. Israel, Finland, USA 2014 [20]	✓	✓	△	✓	✓	✓	✗	✓	✗	✓	✓
Cross et al. Norway 2023 [21]	✓	✓	△	✓	✓	✓	✗	✓	✗	✓	✓
Karagianni et al. Greece 2020 [22]	✓	✓	△	✓	✓	✓	✗	✓	✗	✓	✓
Sembler-Møller et al. Denmark 2020 [23]	✓	✓	△	✓	✓	✓	✗	✓	✗	✓	✓
Chiang et al. USA, Denmark, Korea 2024 [24]	✓	✓	△	✓	✓	✓	✗	✓	✗	✓	✓

Study	clearly stated research question or objective	clearly defined study population	sample size justification	groups recruitment from the same population	valid inclusion and exclusion criteria	cases differentiated from controls	randomization	clearly defined measures	blinded participant status	adjusted statistical methods	summary quality score
Li et al. 2022 China [25]	✓	✓	△	✓	✓	✓	✗	✓	✗	✓	✓
Moreno-Quispe et al. Spain, Peru 2020 [26]	✓	✓	△	✓	✓	✓	✗	✓	✗	✓	✓
Jin et al. China 2019 [27]	✓	✓	△	✓	✓	✓	✗	✓	✗	✓	✓
Lee et al. Korea 2019 [28]	✓	✓	△	✓	✓	✓	△	✓	✗	✓	✓
Sandhya et al. India 2017 [29]	✓	✓	△	✓	✓	✓	✗	✓	✗	✓	✓
Garreto et al. Brazil 2021 [30]	✓	✓	△	✓	✓	✓	✗	✓	✗	✓	✓
Wei et al. China 2020 [31]	✓	✓	△	✓	✓	✓	✗	✓	✗	✓	✓

The table summarizes the methodological quality assessment of all included studies across eleven domains: research question clarity, definition of the study population, sample size justification, recruitment comparability, inclusion/exclusion criteria, differentiation of cases and controls, randomization, measurement validity, blinding, and adequacy of statistical methods.

Each domain was rated according to the ROBVIS tool: Quality assessment, including the main potential risk of bias (https://www.riskofbias.info/welcome/robvis-visualization-tool.

Legend: ✓ for low risk of bias, △ for some concerns, ✗for high risk of bias.

References

[1] G. Piacenza Florezi, F. Pereira Barone, M. A. Izidoro, J. M. Soares-Jr, C. M. Coutinho-Camillo, and S. V. Lourenço, "Targeted saliva metabolomics in Sjögren's syndrome," *Clinics*, vol. 79, Jan. 2024, doi: 10.1016/j.clinsp.2024.100459.

[2] B. Vyas *et al.*, "Raman hyperspectroscopy of saliva and machine learning for Sjögren's disease diagnostics," *Sci Rep*, vol. 14, no. 1, Dec. 2024, doi: 10.1038/s41598-024-59850-6.

[3] A. Alt-Holland *et al.*, "Identification of Salivary Metabolic Signatures Associated with Primary Sjögren's Disease," *Molecules*, vol. 28, no. 15, Aug. 2023, doi: 10.3390/molecules28155891.

[4] P. Bosman *et al.*, "Identification of potential salivary biomarkers for Sjögren's syndrome with an untargeted metabolomic approach," *Metabolomics*, vol. 19, no. 9, Sep. 2023, doi: 10.1007/s11306-023-02040-8.

[5] G. Setti *et al.*, "Metabolic Profile of Whole Unstimulated Saliva in Patients with Sjögren's Syndrome," *Metabolites*, vol. 13, no. 3, Mar. 2023, doi: 10.3390/metabo13030348.

[6] Z. Li *et al.*, "Analysis of the saliva metabolic signature in patients with primary Sjögren's syndrome," *PLoS One*, vol. 17, no. 6 June, Jun. 2022, doi: 10.1371/journal.pone.0269275.

[7] M. Herrala *et al.*, "Variability of salivary metabolite levels in patients with sjögren's syndrome," *J Oral Sci*, vol. 63, no. 1, pp. 22–26, 2021, doi: 10.2334/josnusd.19-0504.

[8] A. Tvarijonaviciute, C. Zamora, S. Martinez-Subiela, F. Tecles, F. Pina, and P. Lopez-Jornet, "Salivary adiponectin, but not adenosine deaminase, correlates with clinical signs in women with Sjögren's syndrome: a pilot study," *Clin Oral Investig*, vol. 23, no. 3, pp. 1407–1414, Mar. 2019, doi: 10.1007/s00784-018-2570-3.

[9] G. Kageyama *et al.*, "Metabolomics analysis of saliva from patients with primary Sjögren's syndrome," *Clin Exp Immunol*, vol. 182, no. 2, pp. 149–153, Nov. 2015, doi: 10.1111/cei.12683.

[10] Y.-C. Tian *et al.*, "Data-Independent Acquisition-Based Quantitative Proteomic Analysis Reveals Potential Salivary Biomarkers of Primary Sjögren's Syndrome," *Chinese Medical Sciences Journal*, vol. 39, no. 1, pp. 19–28, 2024, doi: 10.24920/004338.

[11] N. Di Giorgi *et al.*, "Salivary Proteomics Markers for Preclinical Sjögren's Syndrome: A Pilot Study," *Biomolecules*, vol. 12, no. 6, Jun. 2022, doi: 10.3390/biom12060738.

[12] F. Finamore *et al.*, "Characterization of extracellular vesicle cargo in Sjögren's syndrome through a swath-ms proteomics approach," *Int J Mol Sci*, vol. 22, no. 9, May 2021, doi: 10.3390/ijms22094864.

[13] L. A. Aqrawi, J. L. Jensen, S. Fromreide, H. K. Galtung, and K. Skarstein, "Expression of NGAL-specific cells and mRNA levels correlate with inflammation in the salivary gland, and its overexpression in the saliva, of patients with primary Sjögren's syndrome," *Autoimmunity*, pp. 333–343, 2020, doi: 10.1080/08916934.2020.1795140.

[14] X. Chen *et al.*, "Elevated cytokine levels in tears and saliva of patients with primary Sjögren's syndrome correlate with clinical ocular and oral manifestations," *Sci Rep*, vol. 9, no. 1, Dec. 2019, doi: 10.1038/s41598-019-43714-5.

[15] A. Cecchettini *et al.*, "Phenotyping multiple subsets in Sjögren's syndrome: A salivary proteomic SWATH-MS approach towards precision medicine," *Clin Proteomics*, vol. 16, no. 1, Jun. 2019, doi: 10.1186/s12014-019-9245-1.

[16] L. A. Aqrawi *et al.*, "Proteomic and histopathological characterisation of sicca subjects and primary Sjögren's syndrome patients reveals promising tear, saliva and extracellular vesicle disease biomarkers," *Arthritis Res Ther*, vol. 21, no. 1, Jul. 2019, doi: 10.1186/s13075-019-1961-4.

[17] F. Garza-García, G. Delgado-García, M. Garza-Elizondo, L. Á. Ceceñas-Falcónc, D. Galarza-Delgado, and J. Riega-Torres, "Salivary B2-microglobulin positively correlates with ESSPRI in patients with primary Sjögren's syndrome," *Rev Bras Reumatol*, vol. 57, no. 2, pp. 182–184, 2017, doi: 10.1016/j.rbre.2016.11.001.

[18] L. A. Aqrawi *et al.*, "Identification of potential saliva and tear biomarkers in primary Sjögren's syndrome, utilising the extraction of extracellular vesicles and proteomics analysis," *Arthritis Res Ther*, vol. 19, no. 1, Jan. 2017, doi: 10.1186/s13075-017-1228-x.

[19] N. Delaleu, P. Mydel, J. G. Brun, M. V. Jonsson, A. Alimonti, and R. Jonsson, "Sjögren's syndrome patients with ectopic germinal centers present with a distinct salivary proteome," *Rheumatology (United Kingdom)*, vol. 55, no. 6, pp. 1127–1137, Jun. 2016, doi: 10.1093/rheumatology/kew013.

[20] O. Deutsch *et al.*, "Identification of Sjögren's syndrome oral fluid biomarker candidates following high-abundance protein depletion," *Rheumatology (United Kingdom)*, vol. 54, no. 5, pp. 884–890, Apr. 2014, doi: 10.1093/rheumatology/keu405.

[21] T. Cross *et al.*, "Non-Coding RNA in Salivary Extracellular Vesicles: A New Frontier in Sjögren's Syndrome Diagnostics?," *Int J Mol Sci*, vol. 24, no. 17, Sep. 2023, doi: 10.3390/ijms241713409.

[22] P. Karagianni, A. V. Goules, and A. G. Tzioufas, "Epigenetic alterations in Sjögren's syndrome patient saliva," *Clin Exp Immunol*, vol. 202, no. 2, pp. 137–143, Nov. 2020, doi: 10.1111/cei.13492.

[23] M. L. Sembler-Møller, D. Belstrøm, H. Locht, and A. M. L. Pedersen, "Distinct microRNA expression profiles in saliva and salivary gland tissue differentiate patients with primary Sjögren's syndrome from non-Sjögren's sicca patients," *Journal of Oral Pathology and Medicine*, vol. 49, no. 10, pp. 1044–1052, Nov. 2020, doi: 10.1111/jop.13099.

[24] S. Chiang *et al.*, "Distinctive profile of monomeric and polymeric anti-SSA/Ro52 immunoglobulin A1 isoforms in saliva of patients with primary Sjögren's syndrome and Sicca," *RMD Open*, vol. 10, no. 2, Apr. 2024, doi: 10.1136/rmdopen-2023-003666.

[25] P. Li, Y. Jin, R. Zhao, Z. Xue, and J. Ji, "Expression of ICOS in the salivary glands of patients with primary Sjogren's syndrome and its molecular mechanism," *Mol Med Rep*, vol. 26, no. 5, Nov. 2022, doi: 10.3892/mmr.2022.12864.

[26] L. A. Moreno-Quispe *et al.*, "Association of salivary inflammatory biomarkers with primary Sjögren's syndrome," *Journal of Oral Pathology and Medicine*, vol. 49, no. 9, pp. 940–947, Oct. 2020, doi: 10.1111/jop.13070.

[27] Y. Jin *et al.*, "Tissue-Specific Autoantibodies Improve Diagnosis of Primary Sjögren's Syndrome in the Early Stage and Indicate Localized Salivary Injury," *J Immunol Res*, vol. 2019, 2019, doi: 10.1155/2019/3642937.

[28] J. Lee *et al.*, "Soluble siglec-5 is a novel salivary biomarker for primary Sjogren's syndrome," *J Autoimmun*, vol. 100, pp. 114–119, Jun. 2019, doi: 10.1016/j.jaut.2019.03.008.

[29] P. Sandhya *et al.*, "Diagnostic accuracy of salivary and serum-free light chain assays in primary Sjögren's syndrome: a pilot study," *Int J Rheum Dis*, vol. 20, no. 6, pp. 760–766, Jun. 2017, doi: 10.1111/1756-185X.12965.

[30] L. Garreto *et al.*, "Mapping Salivary Proteases in Sjögren's Syndrome Patients Reveals Overexpression of Dipeptidyl Peptidase-4/CD26," *Front Immunol*, vol. 12, Jun. 2021, doi: 10.3389/fimmu.2021.686480.

[31] P. Wei, Y. Xing, B. Li, F. Chen, and H. Hua, "Proteomics-Based Analysis Indicating α-Enolase as a Potential Biomarker in Primary Sjögren's Syndrome," *Gland Surg*, vol. 9, no. 6, pp. 2054–2063, Dec. 2020, doi: 10.21037/GS-20-814.
