# Supplementary material for: Salivary Biomarkers for the Diagnosis of Sjögren’s Syndrome: A Review of the Last Decade
Source: Biomedicines. 2025 Oct 30;13(11):2664. doi: 10.3390/biomedicines13112664 (PMC12649840; doi:10.3390/biomedicines13112664)
Supplement: Supplementary file 1 [file biomedicines-13-02664-s001.zip › Table S1. Diagnostic criteria of Sjogren syndrome.pdf]

Diagnostic Criteria of Sjögren's Syndrome

**2002 American-European Consensus Group (2002 AECG criteria)** [1]	
**Items**	**1. Ocular dryness symptoms**<br>at least one positive response of the following questions:<br>• Have you had daily, persistent, troublesome dry eyes for more than 3 months?<br>• Do you have a recurrent sensation of sand or gravel in the eyes?<br>• Do you use tear substitutes more than 3 times a day?<br>**2. Oral dryness symptoms**<br>at least one positive response of the following questions:<br>• Have you had a daily feeling of dry mouth for more than 3 months?<br>• Have you had recurrently or persistently swollen salivary glands as an adult?<br>• Do you frequently drink liquids to aid in swallowing dry food?<br><br>**3. Ocular signs - at least one of the following two tests:**<br>• Schirmer's I test, performed without anesthesia (<= 5 mm in 5 minutes)<br>• Rose bengal score or other ocular dye score (>=4 according to van Bijsterveld's scoring system)<br>**4. Histopathology:**<br>In minor salivary glands (obtained through normal-appearing mucosa) focal lymphocytic sialoadenitis, evaluated by an expert histopathologist, with a focus score >=1, defined as a number of lymphocytic foci (which are adjacent to normal-appearing mucous acini and contain more than 50 lymphocytes) per 4 mm2 of glandular tissue<br>**5. Salivary gland involvement- at least one of the following diagnostic tests:**<br>• Unstimulated whole salivary flow (<1.5 ml in 15 minutes)<br>• Parotid sialography showing the presence of diffuse sialectasias (punctate, cavitary or destructive pattern), without evidence of obstruction in the major ducts<br>• Salivary scintigraphy showing delayed uptake, reduced concentration and/or delayed excretion of tracer20<br>6. Autoantibodies:<br>• Antibodies to Ro(SSA) or La(SSB) antigens, or both.
**Rules for classification**	For primary SS<br>In patients without any potentially associated disease, primary SS may be defined as follows:<br>• The presence of any 4 of the 6 items is indicative of primary SS, as long as either item 4 (Histopathology) or 6 (Serology) is Positive<br>• The presence of any 3 of the 4 objective criteria items (items 3-6)<br>For secondary SS<br>In patients with a potentially associated disease (for instance, another well-defined connective tissue disease), the presence of item 1 or item 2 plus any 2 from among items 3,4, and 5 may be considered as indicative of secondary SS
**Exclusion criteria:**	Past head and neck radiation treatment<br>Hepatitis C infection<br>Acquired immunodeficiency disease (AIDS)<br>Pre-existing lymphoma<br>Sarcoidosis<br>Graft versus host disease<br>Use of anticholinergic drugs (since a time shorter than 4-fold the half-life of the drug)

American College of Rheumatology Classification Criteria 2012 (ACR Criteria 2012) [2]<br>A patient is classified as having Sjögren's syndrome if they meet at least 2 of the 3 following criteria.	
**1**	Positive serum anti-SSA/Ro and/or anti-SSB/La or (positive rheumatoid factor and ANA titer ≥1:320)
**2**	Keratoconjunctiviti ssicca with ocular staining score 3 (assuming that individual is not currently using daily eyedrops for glaucoma and has not had corneal surgery or cosmetic eyelid surgery in the last 5 years)
**3**	Labial salivary gland biopsy exhibiting focal lymphocytic sialadenitis with a focus score ≥1 focus/4mm2
**Exclusion criteria:**	History of head and neck radiatiotreatment<br>Hepatitis C infection<br>Acquired immunodeficiency syndrome<br>Sarcoidosis<br>Amyloidosis<br>Graft versus host disease<br>IgG4-related disease+

2016 American College of Rheumatology (ACR)/European League Against Rheumatism (EULAR) (2016 ACR/EULAR criteria) [3]		
	**A patient is classified as having Sjögren's syndrome if the total score is ≥4**	**Weight**
**Items**	1. Labial salivary gland with focal lymphocytic sialadenitis and focus score of ≥ 1 foci/4 mm^2	**3**
	2. Anti-SSA/Ro-positive	**3**
	3. Ocular Staining Score ≥ 5 (or van Bijsterveld score ≥ 4) in at least one eye [4]	**1**
	4. Schirmer's test ≤ 5 mm/5 min in at least one eye	**1**
	5. Unstimulated whole saliva flow rate ≤ 0.1 mL/min [5]	**1**
**Inclusion criteria***		
	Any patient with at least 1 symptom of ocular or oral dryness, defined as a positive response to at least 1 of the following questions:<br>1) Have you had daily, persistent, troublesome dry eyes for more than 3 months?<br>2) Do you have a recurrent sensation of sand or gravel in the eyes?<br>3) Do you use tear substitutes more than 3 times a day?<br>4) Have you had a daily feeling of dry mouth for more than 3 months?<br>5) Do you frequently drink liquids to aid in swallowing dry food?<br><br>* Patients who are normally taking anticholinergic drugs should be evaluated for objective signs of salivary hypofunction and ocular dryness after a sufficient interval without these medications in order for these components to be a valid measure of oral and ocular dryness.	
**Exclusion criteria**	1) history of head and neck radiation treatment,<br>2) active hepatitis C infection (with confirmation by polymerase chain reaction,<br>3) AIDS,<br>4) sarcoidosis,<br>5) amyloidosis,<br>6) graft-versus-host disease,<br>7) IgG4-related disease.	

Comparison of Criteria			
Criteria	**AECG (2002)** [1]	**ACR (2012)**[2]	**ACR/EULAR (2016)**[3]
**Oral Symptoms**	Required	Not required	Included
**Ocular Symptoms**	Required	Included	Included
**Schirmer's Test**	≤5 mm/5 minutes	Not required	≤5 mm/5 minutes (1 point)
**Ocular Staining Score (OSS)**	≥4 (van Bijsterveld)	≥3	≥5 (1 point)
**Salivary Gland Biopsy**	Focus score ≥1	Focus score ≥1	Focus score ≥1 (3 points)
**Salivary Flow Rate**	≤1.5 mL/15 minutes	Not required	≤0.1 mL/minute (1 point)
**Autoantibodies**	Anti-SSA/SSB	Anti-SSA	Anti-SSA (3 points)
**Classification**	4/6 criteria	2/3 criteria	Score ≥4

Table S1: Diagnostic criteria for Sjögren's syndrome according to the 2002 American–European Consensus Group (AECG), 2012 American College of Rheumatology (ACR), and 2016 American College of Rheumatology/European League Against Rheumatism (ACR/EULAR). The table summarizes the major classification systems used for diagnosing Sjögren's syndrome, including required symptoms, ocular and salivary tests, histopathological findings, and autoantibody profiles. Comparative differences in inclusion/exclusion criteria and scoring thresholds are presented.

Reference

[1] I. A. Leyla Y. Teos, "Classification criteria for Sjögren's syndrome: a revised version of the European criteria proposed by the American-European Consensus Group," 2002. [Online]. Available: http://ard.bmj.com/

[2] S. C. Shiboski *et al.*, "American College of rheumatology classification criteria for Sjögren's syndrome: A data-driven, expert consensus approach in the Sjögren's International Collaborative Clinical Alliance cohort," *Arthritis Care Res (Hoboken)*, vol. 64, no. 4, pp. 475–487, Apr. 2012, doi: 10.1002/acr.21591.

[3] C. H. Shiboski *et al.*, "2016 American College of Rheumatology/European League Against Rheumatism Classification Criteria for Primary Sjögren's Syndrome: A Consensus and Data-Driven Methodology Involving Three International Patient Cohorts," *Arthritis and Rheumatology*, vol. 69, no. 1, pp. 35–45, Jan. 2017, doi: 10.1002/art.39859.

[4] R. Seror and S. Bowman, "Outcome Measures in Primary Sjögren's Syndrome," *Arthritis Care Res (Hoboken)*, vol. 72, no. S10, pp. 134–149, Oct. 2020, doi: 10.1002/acr.24331.

[5] M. Navazesh and S. K. S. Kumar, "Measuring salivary flow," *The Journal of the American Dental Association*, vol. 139, pp. 35S-40S, May 2008, doi: 10.14219/jada.archive.2008.0353.
