# Supplementary material for: Salivary Biomarkers for the Diagnosis of Sjögren’s Syndrome: A Review of the Last Decade
Source: Biomedicines. 2025 Oct 30;13(11):2664. doi: 10.3390/biomedicines13112664 (PMC12649840; doi:10.3390/biomedicines13112664)
Supplement: Supplementary file 1 [file biomedicines-13-02664-s001.zip › Table S2. Results.pdf]

**Results**

**(A) Metabolomics analysis**

| Author, Setting Year, | Study Group | Healthy Control (HC) or other | Diagnostic criteria for Sjögren's syndrome | Sample | Analytical Methods | Results |
|---|---|---|---|---|---|---|
| Piacenza Florezi et al. Brazil 2024 [1] | 10 pSjS 8♀ 2♂ Average age 41 | 10 HC 9♀ 1♂ Average age 46.8 | 2002 AECG criteria | NWS | Ultra-high Performance Liquid Chromatograph Coupled Mass Spectrometry (UPLC MS) | ↑ concentration in pSS ($p<0.05$.) Guanosine p= 0.011 L-Lactic acid p= 0.024 Malic acid p= 0.032 L-Arginine p= 0.010 L-Threonine p= 0.045 L-Valine p= 0.027 4-Hydroxyproline p= 0.003 L-Leucine p= 0.011 L-Methionine p= 0.032 L-Alanine p= 0.019 L-Isoleucine p= 0.005 |
| Vyas et al. USA 2024 [2] | 26-SjS 4♂ 22♀ Age 60 (± 10) | 24-HC 14♂ 8♀ Age 60 (± 10) 22-RD (radiation therapy for head and neck cancers) 18♂ 4♀ Age 66 (± 9) | Not specified in the source article | NWS | Raman hyperspectroscopy, machine learning | SjS vs HC & RD Proline ↓ Tryptophan ↓ |
| Alt-Holland et al. USA 2023 [3] | 30 pSjS 28♀ 2♂ Age 62,2 +- 10,2 | 30 HC 21♀ 9♂ Age 37,2 +-15,6 | ACR/EULAR 2016 criteria | NWS | NMR spectroscopy | ↑ pSjS: glucose (p= 0.0289) glycerol (p= 0.0302) lactate (p=0.0074) taurine (p=0.0001) ↓ pSjS: 5-ANP (p=0.0027) acetate (p=0.0043) butyrate (p=0.0048) fucose (p=0.0117) propionate (p=0.0027) |
| Alt-Holland et al. USA 2023 [3] | 24 pSjS 22♀ 2♂ Age 60,5 (iqr 10,5) | 14 HC 11♀ 3♂ Age 49,5 (iqr 28) | ACR/EULAR 2016 criteria | NWS | NMR spectroscopy | ↑ pSjS: choline (p=0.0191) glucose (p= 0.0194) glycerol (p= 0.0148) lactate (p=0.0559) taurine (p=0.0041) fructose (p=0.0544) ↓ pSjS: 5-ANP (p=0.0034) acetate (p=0.0148) butyrate (p=0.0041) propionate (p=0.0003) |
| Bosman et al. | 18 SjS ♀ | 22 HC ♀ | 2002 AECG criteria | NWS, SWS | LC-HRMS | pSjS vs HC Alanine (AUC): 0.873. |

| Author, Setting Year, | Study Group | Healthy Control (HC) or other | Diagnostic criteria for Sjögren's syndrome | Sample | Analytical Methods | Results |
|---|---|---|---|---|---|---|
| France, Brasil 2023 [4] | Age 35 to 82 years old (53.4 ± 12.1) | Age 55 to 72 years old (60.0 ± 4.5) | | | | Succinic Acid: AUC: 0.906.<br>Isovaleric Acid: AUC: 0.871<br><br>Amino acids or derivatives: Alanine, serine, threonine, leucine/isoleucine, lysine, phenylalanine, glutamic acid ↓<br><br>Purine derivatives: Hypoxanthine, uric acid ↓<br><br>Organic acids: Lactic acid, malic acid ↓<br>Carboxylic acids: Succinic acid, isovaleric acid ↓<br><br>SjS impact on metabolic pathways:<br><br>Amino Acid Metabolism: glutamic acid and lysine ↓<br><br>Purine Metabolism: hypoxanthine and uric acid ↓<br><br>Citric Acid Cycle: succinic acid and malic acid ↓<br><br>Threitol ↓<br>D-$\alpha$-Aminobutyric acid ↓<br>GABA ↓ |
| Setti et al. Italy 2023 [5] | 7 pSjS ♀ | 6 HC♀ | ACR/EULAR 2016 criteria | NWS, | 1D 1H-NMR CPMG NMR spectra, PCA, PLS-DA | 50 metabolites<br><br>SjS methylamine ↑<br>$p < 0.05$<br><br>HC proline ↑<br>$p < 0.05$ |
| Li et al. 2022 China [6] | 32 pSjS (test group 20, validation group 12) | 38 HC (test group 25, validation group 13) | ACR/EULAR 2016 criteria | NWS | UPLC-HRMS | pSjS:<br>Significant enrichment ($p < 0.05$) of metabolism of tryptophan, tyrosine, aspartate, asparagine and carbon fixation.<br><br>Metabolites involved in metabolism of tryptophan, aspartate and asparagine were upregulated ↑<br><br>Metabolites derived from amino acids was upregulated ↑: phenylalanyl-alanine, tryptophyl-isoleucine, tyrosyl-phenylalanine, asparaginyl-valine, |

| Author, Setting Year, | Study Group | Healthy Control (HC) or other | Diagnostic criteria for Sjögren's syndrome | Sample | Analytical Methods | Results |
|---|---|---|---|---|---|---|
| | | | | | | aspartyl-isoleucine and tyrosyl-hydroxyproline.<br><br>Purine metabolites: 8-hydroxyadenine and oxypurinol<br><br>Metabolites involved in carbon fixation ↓<br><br>38 metabolites diagnostic value, with an AUC above 0.7<br><br>30 metabolites diagnostic value, with an AUC above 0.8<br><br>Aspartyl-isoleucine (p<0.001)<br><br>Phenylalanyl-alanine |
| Herrala et al.<br>Finland<br>2021<br>[7] | 14 ♀ pSjS (4x14=56 samples) | 15 HC | 2002 AECG criteria | SWS | proton nuclear magnetic resonance (1H-NMR) spectroscopy | pSjS:<br>Choline ↑ (higher at each time: p = 0.015, 0.023, 0.001, <0.001)<br><br>Taurine ↑ (higher at the three last points p = 0.023, 0.002, 0.001),<br><br>Alanine ↑ (higher at the two last points (P = 0.005 and 0.001)<br><br>Glycine ↑ (higher at the three last points (p = 0.040, 0.032, 0.004) |
| Tvari-jonaviciute et al. 2019<br>Spain<br>[8] | SjS 17♀<br><br>non-SS sicca group 19♀ | 13 HC ♀ | 2002 AECG criteria | NWS | ELISA | SjS:<br>Salivary adiponectin ↑<br><br>SjS and non-SS sicca:<br><br>Adenosine deaminase (ADA) ↑ |
| Kageyama et al.<br>Japan<br>2015<br>[9] | 12♀ pSjS mean age 44.26±13.01 | 21 HC ♀<br>Age matched | 2002 AECG criteria | NWS | Principal component analysis (PCA) | pSjS<br>88 metabolites were detected (41 ↓)<br>pSjS<br>glycine ↓<br>tyrosine ↓<br>uric acid ↓<br>fucose ↓ |

**(B) Salivary Proteomics**

| **Author, Setting Year,** | **Study Group** | **Healthy Control (HC) or other** | **Diagnostic criteria for Sjögren's syndrome** | **Sample** | **Analytical Methods** | **Results** |
|---|---|---|---|---|---|---|
| Tian et al. China 2024 [10] | 36 pSS Discovery 24(21♀) Age 41.1±10.9<br><br>Validation 12 (10♀) Age 40.6 ±12.8 | 38 HC Discovery 25(23♀) 41.4±12.2<br><br>Validation 13 (12♀), 44.1±127 | ACR/EULAR 2016 criteria | NWS | Liquid chromatography mass spectrometry (LC- MS/MS)<br><br>data- independent acquisition-mass spectrometry (DIA- MS) mode | 19 proteins – potential biomarkers: Q13217, P01034, P07711, P09958, P28065, 043852, 043505, P25789, Q9UBX7, Q9NRI3, P35030, P12109, O75976, Q92743, P16870, P13798, P01011, P32320, P08670<br><br>Up-regulated in pSjS: CA-I the most up-regulated (fold change = 10)<br><br>B2 microglobulin, S100 A8, S100A11.-(2- or 3-fold changes)<br><br>HTRA-1 (Q92743)<br><br>down-regulated in pSjS Kallikreins KLKs |
| Giorgi et al. 2022 Italy [11] | pSjS 11<br><br>preclinical SjS SSA+ 8 | 8 HC age,gender-matched | ACR/EULAR 2016 criteria | NWS , EVs | high-performance liquid chromatography–tandem mass spectrometry (HPLC-MS/MS) | pSjS 11and preclinical SjS SSA+ compared to HC<br><br>MUC5B ↓<br><br>Prolactin-Inducible Protein (PIP) ↓<br><br>CST4 ↓<br><br>lipocalin 1 ↓ |
| Finamore et al. 2021 Italy [12] | 7 pSjS ♀ age 25–75 | 5 HC ♀ age 26–65 | ACR/EULAR 2016 criteria | NWS, EVs | SWATH-MS | pSjS EVs vs NWS increase of:<br><br>S100 protein family (S100A7, A8, A9, A11, A12),<br><br>resistin (RETN),<br><br>serpin peptidase inhibitors (SERPINB1, SEPINB5),<br><br>azurocidin (AZU1),<br><br>monocyte differentiation antigen (CD14)<br><br>annexin A2 (ANXA2),<br><br>cofilin-1 (CFL-1),<br><br>plastin-2 (LCP1),<br><br>macrophage migration inhibitory factor (MIF) |

| Author, Setting Year, | Study Group | Healthy Control (HC) or other | Diagnostic criteria for Sjögren's syndrome | Sample | Analytical Methods | Results |
|---|---|---|---|---|---|---|
| Aqrawi et al. Norway 2020 [13] | 27 pSjS | 32 HC age- and gender matched | 2002 AECG criteria | SWS, EVs | LC-MS | pSjS ↑ Neutrophil gelatinase-associated lipocalin (NGAL) |
| Chen et al. Norway 2019 [14] | pSjS 29 ♀ age 56.8 ± 13.0 years<br><br>20 non-SjS (sicca control) age 51.7 ± 10.6 years | 17 HC ♀ age 45.4 ± 10.9 years | 2002 AECG criteria | NWS, SWS | immunoassay technology (Bio-Plex XMap; Bio-Rad Laboratories, Inc., Hercules, CA, USA) | pSjS ↑ IP-10<br><br>pSjS, non-SjS ↑ MIP-1$\alpha$ |
| Cecchettini et al. 2019 Italy [15] | SjS 20 | 20 HC age- and sex-matched | 2002 AECG criteria | NWS | nano-HPLC-SWATH-MS, ELISA | 203(proteins differently expressed in pSjS)<br><br>SjS:<br>prolactin-inducible protein PIP protein ↓<br>proline-rich proteins (PRPs) ↓<br>cystatins ↓ (except for cystatin A and B)<br>carboanhydrase VI ↓<br><br>cathepsin family ↑<br><br>S100A proteins ↑ |
| Aqrawi et al. Norway 2019 [16] | 10 pSjS ♀<br><br>15 non-SjS sicca | 10 HC | 2002 AECG criteria | SWS, EVs | LC-MS | SWS Non-SS vs. pSS↑<br><br>peptidyl-prolyl cis-trans isomerase FKBP1A (FKBP1A) ↑<br><br>CD44 antigen (CD44) ↑<br>Beta-2-microglobulin ↑<br><br>SWS HC vs. pSS↑<br><br>Beta-2-microglobulin ↑<br>Secreted Ly-6/uPAR-related protein 1 SLUR1 ↑<br>Clusterin CLUS ↑<br><br>EVs SWS Non-SS vs. pSS↑<br><br>CD44 CD44 antigen ↑<br>MVP Major vault protein ↑<br>NGAL Neutrophil gelatinase-associated lipocalin ↑<br><br>EVs SWS HC vs. pSS↑<br><br>FCN1 Ficolin-1 ↑<br>CD44 CD44 antigen ↑<br>ANXA4 Annexin A4 ↑ |

| Author, Setting Year, | Study Group | Healthy Control (HC) or other | Diagnostic criteria for Sjögren's syndrome | Sample | Analytical Methods | Results |
|---|---|---|---|---|---|---|
| Garza-García et al.<br>Mexico<br>2017<br>[17] | 71 ♀pSjS<br><br>age 53.05 ± 12.19 years<br><br>PASS(Patient-Acceptable Symptom State) (n = 28) | non-PASS groups (n = 43) | 2002 AECG criteria | NWS | Not specified in the source article | ↑ β2-microglobulin correlation with ↑ ESSPRI (EULAR Sjögren's syndrome Patient Reported Index) |
| Aqrawi et al.<br>Norway<br>2017<br>[18] | 27 pSjS | 32 HC age- and gender matched | AECG criteria 2002 | SWS, EVs | LC-MS Liquid chromatography-mass spectrometry and size exclusion chromatography | SWS pSjS ↑<br><br>LCN2<br>GRN<br>CALM<br>NPC2<br>CALML5<br><br>EVs pSjS ↑<br>APMAP<br>GNA13<br>WDR1<br>SIRPA<br>LSP1 |
| Delaleu et al.<br>Switzerland<br>2016<br><br>[19] | 48 ♀ pSjS<br><br>24 Hyposalivation<br>24 Normal<br><br>GC (ectopic germinal centers) + 34<br>GC - 14 | ------ | 2002 AECG criteria | NWS | Human Discovery MAP 1.0. | Hyposalivation:<br><br>uromodulin UMOD ↓<br>secreted phosphoprotein 1 (SPP1) ↓<br><br>GC +<br>pregnancy-associated plasma protein A (PAPPA) $p=6.795x10^{-08}$ ↑<br><br>Thrombospondin 1 THBS1 $p=5.178 x10^{-04}$ ↑<br><br>uromodulin UMOD $p=2.194x 10^{-05}$ ↓<br><br>IL5 $p=1.959 x 10^{-04}$ ↓ |
| Deutsch et al.<br>Israel, Finland, USA<br>2014<br>[20] | 18 SjS ♀<br>Age: 55.5 years (s.d. 15.3) | 18 HC ♀<br>[52.1 years (s.d. 7.2) | 2002 AECG criteria | NWS | LC-MS/MS | 79 biomarker candidates pSjS<br><br>strongly differentially:<br>profilin↑<br>Histone H2B ↑<br>Carcinoembryonic antigen ↑<br>MMP 9 ↑<br>MPO precursor ↑<br><br>CA-I ↓ |

| Author, Setting Year, | Study Group | Healthy Control (HC) or other | Diagnostic criteria for Sjögren's syndrome | Sample | Analytical Methods | Results |
|---|---|---|---|---|---|---|
| | | | | | | bacterial permeability increasing (BPI) protein ↓ |

**(C) Molecular Biomarkers**

| Author, Setting Year, | Study Group | Healthy Control (HC) or other | Diagnostic criteria for Sjögren's syndrome | Sample | Analytical Methods | Results |
|---|---|---|---|---|---|---|
| Cross et al.<br>Norway<br>2023<br>[21] | pSjS 11♀<br><br>mean age of 55 | 11 HC ♀<br><br>mean age of 55 | ACR/EULAR 2016 criteria | SWS, EVs | Qiagen exoRNeasy Midi Kit, Affymetrix Clariom D™ microarrays | 1475 transcripts (notable difference in abundance between the pSjS and HC)<br><br>pSjS tRNAs ↓<br><br>tRNA-Ile-AAT-2-1 (biomarker candidate) |
| Karagianni et al.<br>Greece<br>2020<br>[22] | 16 SjS patients age = 63·2, mean ± standard deviation (s.d.) = 8·8 | 10 sicca controls with negative MSG biopsy<br>age = 57·2, mean ± s.d. = 13 ·7 | 2002 AECG criteria | NWS | PCR | Statistically significant reduction in H19 ICR methylation in SjS patients' saliva vs. sicca controls<br><br>Significant negative correlation between C4 serum levels and H19 ICR methylation |
| Sembler-Møller et al.<br>Denmark<br>2020<br>[23] | pSjS 24<br>22♀ 2♂<br>age 55 ± 11 | 16 non-Sjögren's sicca patients<br>14♀ 2♂<br>age 53 ± 16 | ACR/EULAR 2016 criteria | NWS, SWS | miRCURY LNA™ Universal RT miRNA PCR, Polyadenylation, cDNA synthesis kit (Exiqon) | pSjS<br>14 miRNAs differed significantly<br><br>let-7i-5p ↑ p=0.00006<br>miR-17-5p ↓ p=0.00003<br>miR-328-3p ↓ p= 0.00024<br>miR-7-5p ↓ p=0.00149<br>miR-30c-5p ↓ p=0.00173<br>miR-191-5p ↓ p=0.00199<br>miR-106a-5p ↓ p=0.00202<br>miR-222-3p ↑ p=0.00203<br>miR-154-5p ↓ p=0.00206<br>miR-215-5p ↓ p=0.00207<br>miR-106b-5p ↓ p=0.00229<br>let-7e-5p ↑ p=0.0023<br>miR-409-3p ↓ p=0.00297<br>miR-20b-5p ↓ p=0.00359 |

**(D) Autoimmune Biomarker Panel**

| Author, Setting Year, | Study Group | Healthy Control (HC) or other | Diagnostic criteria for Sjögren's syndrome | Sample | Analytical Methods | Results |
|---|---|---|---|---|---|---|
| Chiang et al.<br>USA,<br>Denmark,<br>Korea | ♀ 94%<br>34 pSjS mean age (± SD) | 41 HC | ACR Criteria 2012 | NWS | Electric Field-Induced Release and | pSjS↑<br>anti- SSA/Ro52<br>isotypes IgG/M/A,<br>IgG, IgA, IgA1, IgA2 |

| Author, Setting Year, | Study Group | Healthy Control (HC) or other | Diagnostic criteria for Sjögren's syndrome | Sample | Analytical Methods | Results |
| --- | --- | --- | --- | --- | --- | --- |
| 2024 [24] | 54.6±10.5 years<br><br>35 patients with Sicca mean age 57.3±14.5 years | | | | Measurement (EFIRM) immunoassay | SICCA>pSJS mIgA1<br><br>SICCA<pSJS pIgA1 |
| Li et al. 2022 China [25] | 95 pSjS ♀ 48.82±1.33 | 65 HC ♀ 47.31±1.425 | ACR/EULAR 2016 criteria | NWS | ELISA | ↑ pSjS: ICOS (Inducible T Cell Co-Stimulator) |
| Moreno-Quispe et al. Spain, Peru 2020 [26] | 36 pSjS♀ age of 56.58 ± 12.35 | 35 HC ♀ age of 54.40 ± 9.16 | 2002 AECG criteria | NWS, SWS | high-sensitivity multiplex map human immunoassays HSTCMAG-28SK | pSjS ↑ IL-6 p = 0.0001 |
| Jin et al. China 2019<br><br>[27] | 12 pSjS | 24 HC | 2002 AECG criteria ACR Criteria 2012 | NWS | ELISA | pSjS<br><br>Saliva anti-CA6 IgG ↑<br>Saliva anti-SP1 IgG ↑<br>Saliva anti-PSP IgG ↑ |
| Lee et al. Korea 2019 [28] | 170 pSjS<br><br>validation cohort 45 pSjS | 25 HC<br>78 non SjS sicca patients<br><br>43 patients with systemic lupus erythematosus (SLE)<br><br>validation cohort 45 non SS sicca patients | 2002 AECG criteria<br><br>Validation cohort ACR/EULAR 2016 criteria | NWS | ELISA | siglec-5 p=0.001 significantly higher in pSjS ↑<br><br>validation cohort siglec-5 higher in pSjS ↑ |
| Sandhya et al. India 2017 [29] | 15 pSjS<br>13♀ 2♂<br><br>age 30–43 | 15 HC | 2002 AECG criteria<br><br>ACR Criteria 2012 | NWS | FREELITETM Human Kappa and Lambda Free Kit | pSjS<br><br>salivary kappa (κ) Free Light Chains ↑<br><br>salivary Lambda (λ) Free Light Chains ↑ |

**(E) Enzymatic Markers in Saliva**

| Author, Setting Year, | Study Group | Healthy Control (HC) or other | Diagnostic criteria for Sjögren's syndrome | Sample | Analytical Methods | Results |
| --- | --- | --- | --- | --- | --- | --- |
| Garreto et al. Brazil 2021<br><br>[30] | 10 pSjS<br>10 sSjS<br><br>19♀ 1♂ | 20 HC<br><br>19♀ 1♂ | ACR/EULAR 2016 criteria | SWS | Invitrogen Qubit® 2.0 Fluorometer, Invitrogen™ Qubit™ Protein Assay | pSjS: dipeptidyl peptidase-4/CD26 (DPP4/ CD26) ↑<br><br>sSjS Matrix Metalloproteinase-9 (MMP9) ↑ |

| Author, Setting Year, | Study Group | Healthy Control (HC) or other | Diagnostic criteria for Sjögren's syndrome | Sample | Analytical Methods | Results |
|---|---|---|---|---|---|---|
| | | | | | Kit, ELISA, LC-MS/MS | SjS: Neutrophil Elastase (ELANE) ↑<br>Cathepsin G (CTSG) ↑<br>Myeloblastin (PRTN3) ↑ |
| Wei et al. China 2020 [31] | 10 pSjS | 10 HC | 2002 AECG criteria | Not specified in the source article | LC-MS/MS, Western blot analysis | $\alpha$-enolase ↑ fold change 4,21 in pSjS vs HC |

**Table S3.** (A-E). Summary of salivary biomarkers investigated for the diagnosis of primary Sjögren's syndrome (pSjS), grouped by biomarker category (A–E). For each study, details include author, country, year, diagnostic criteria, saliva type, analytical methods, and key findings. Abbreviations are listed below.

**Abbreviations used in Table S3A-E:**

♂- male; ♀-female; ↑- increase; ↓- decrease; ACR - American College of Rheumatology; ADA - Adenosine Deaminase; ANXA2 - Annexin A2; ANXA4 - Annexin A4; APMAP - Adipocyte Plasma Membrane-Associated Protein; AUC - Area Under the Curve; AZU1 - Azurocidin 1; BPI - Bacterial Permeability-Increasing Protein; CA-I - Carbonic Anhydrase I; CA6 - Carbonic Anhydrase 6; CALM - Calmodulin; CALML5 - Calmodulin-Like 5; CD4+ T - Subpopulation of T-helper lymphocytes; CD14 - Cluster of Differentiation 14; CD44 - CD44 antigen; CEA - Carcinoembryonic Antigen; CFL-1 - Cofilin-1; CLUS - Clusterin; CST4 - Cystatin-S; DIA-MS - Data-Independent Acquisition Mass Spectrometry; DPP4/CD26 - Dipeptidyl Peptidase-4/Cluster of Differentiation 26; EFIRM - Electric Field-Induced Release and Measurement; ELISA - Enzyme-Linked Immunosorbent Assay; ESSDAI - EULAR Sjögren's Syndrome Disease Activity Index; ESSPRI - EULAR Sjögren's Syndrome Patient Reported Index; EVs - Extracellular Vesicles; FGF2 - Basic Fibroblast Growth Factor; FCN1 - Ficolin-1; FLCs - Free Light Chains; FKBP1A - FK506 Binding Protein 1A; G-CSF - Granulocyte Colony-Stimulating Factor; GM-CSF - Granulocyte Macrophage Colony-Stimulating Factor; GNA13 - G Protein Subunit Alpha 13; GRN - Granulin; HC - Healthy Control; H2B - Histone H2B; HLA-DQA1 - HLA Class II Gene, DQA1 Allele; HLA-DQB1 - HLA Class II Gene, DQB1 Allele; HPLC - High-Performance Liquid Chromatography; ICOS - Inducible T-cell CO-Stimulator; IFN-$\gamma$ - Interferon Gamma; IgA - Immunoglobulin A; IgG - Immunoglobulin G; IL-1B - Interleukin-1 beta; IL-1ra - IL-1 Receptor Antagonist; IL-5 - Interleukin-5; IL-6 - Interleukin-6; IL-8 - Interleukin-8; IP-10 - Interferon Gamma-Induced Protein 10; KLKs - Kallikreins; LC-HRMS - Liquid Chromatography-High Resolution Mass Spectrometry; LC-MS/MS - Liquid Chromatography-Tandem Mass Spectrometry; LCN2 - Neutrophil Gelatinase-Associated Lipocalin; LCP1 - Lymphocyte Cytosolic Protein 1; LSP1 - Lymphocyte-Specific Protein 1; MCP-1 - Monocyte Chemoattractant Protein 1; MCTD - Mixed Connective Tissue Disease; MIF - Macrophage Migration Inhibitory Factor; MIP-1$\alpha$/CCL3 - Macrophage Inflammatory Protein 1 Alpha; miRNA - MicroRNA; MMP9 - Matrix Metalloproteinase 9; MPO - Myeloperoxidase; MSG - Minor Salivary Gland; MVP - Major Vault Protein; MUC5B - Mucin 5B; NGAL - Neutrophil Gelatinase-Associated Lipocalin; NMR - Nuclear Magnetic Resonance; NPC2 - Niemann-Pick Disease, Type C2; NWS - Non-Stimulated Whole Saliva; PAPPA - Pregnancy-Associated Plasma Protein A; PASS - Patient-Acceptable Symptom State; PCA - Principal Component Analysis; PCR - Polymerase Chain Reaction; PDGF-BB - Platelet-Derived Growth Factor BB; PIP - Prolactin-Inducible Protein; PLS-DA - Partial Least Squares Discriminant Analysis; PRISMA - Preferred Reporting Items for Systematic Reviews and Meta-Analyses; PRPs - Proline-Rich Proteins; PRTN3 - Myeloblastin; PSP - Parotid Secretory Protein; pSjS - Primary Sjögren's Syndrome; PSS - Primary Sjögren's Syndrome; RANTES - Regulated on Activation, Normal T Cell Expressed and Secreted; RD - Radiation Therapy for Head and Neck Cancers; RETN - Resistin; RZS - Rheumatoid Arthritis; S100A - S100 Calcium-Binding Protein A; S100A8, S100A11 - Proteins from the S100 family; SCC - Squamous Cell Carcinoma; SERPINB1 - Serpin Family B Member 1; SIRPA - Signal-Regulatory Protein Alpha; SLUR1 - Secreted Ly-6/uPAR-Related Protein 1; SP1 - Salivary Protein 1; SPP1 - Secreted Phosphoprotein 1; SSA/Ro - Autoantigen SSA/Ro; sSjS - Secondary Sjögren's Syndrome; SWATH-MS - Sequential Window Acquisition of All Theoretical Fragment Ion Spectra; SWS - Stimulated Whole Saliva; THBS1 - Thrombospondin 1; TNFa - Tumor Necrosis Factor alpha; TNF-$\alpha$ - Tumor Necrosis Factor Alpha; UPLC-MS - Ultra-Performance

Liquid Chromatography-Mass Spectrometry; UMOD - Uromodulin; VEGF - Vascular Endothelial Growth Factor; WDR1 - WD Repeat Domain 1.

**Reference**


[1] G. Piacenza Florezi, F. Pereira Barone, M. A. Izidoro, J. M. Soares-Jr, C. M. Coutinho-Camillo, and S. V. Lourenço, "Targeted saliva metabolomics in Sjögren's syndrome," *Clinics*, vol. 79, Jan. 2024, doi: 10.1016/j.clinsp.2024.100459.

[2] B. Vyas *et al.*, "Raman hyperspectroscopy of saliva and machine learning for Sjögren's disease diagnostics," *Sci Rep*, vol. 14, no. 1, Dec. 2024, doi: 10.1038/s41598-024-59850-6.

[3] A. Alt-Holland *et al.*, "Identification of Salivary Metabolic Signatures Associated with Primary Sjögren's Disease," *Molecules*, vol. 28, no. 15, Aug. 2023, doi: 10.3390/molecules28155891.

[4] P. Bosman *et al.*, "Identification of potential salivary biomarkers for Sjögren's syndrome with an untargeted metabolomic approach," *Metabolomics*, vol. 19, no. 9, Sep. 2023, doi: 10.1007/s11306-023-02040-8.

[5] G. Setti *et al.*, "Metabolic Profile of Whole Unstimulated Saliva in Patients with Sjögren's Syndrome," *Metabolites*, vol. 13, no. 3, Mar. 2023, doi: 10.3390/metabo13030348.

[6] Z. Li *et al.*, "Analysis of the saliva metabolic signature in patients with primary Sjögren's syndrome," *PLoS One*, vol. 17, no. 6 June, Jun. 2022, doi: 10.1371/journal.pone.0269275.

[7] M. Herrala *et al.*, "Variability of salivary metabolite levels in patients with sjögren's syndrome," *J Oral Sci*, vol. 63, no. 1, pp. 22–26, 2021, doi: 10.2334/josnusd.19-0504.

[8] A. Tvarijonaviciute, C. Zamora, S. Martinez-Subiela, F. Tecles, F. Pina, and P. Lopez-Jornet, "Salivary adiponectin, but not adenosine deaminase, correlates with clinical signs in women with Sjögren's syndrome: a pilot study," *Clin Oral Investig*, vol. 23, no. 3, pp. 1407–1414, Mar. 2019, doi: 10.1007/s00784-018-2570-3.

[9] G. Kageyama *et al.*, "Metabolomics analysis of saliva from patients with primary Sjögren's syndrome," *Clin Exp Immunol*, vol. 182, no. 2, pp. 149–153, Nov. 2015, doi: 10.1111/cei.12683.

[10] Y.-C. Tian *et al.*, "Data-Independent Acquisition-Based Quantitative Proteomic Analysis Reveals Potential Salivary Biomarkers of Primary Sjögren's Syndrome," *Chinese Medical Sciences Journal*, vol. 39, no. 1, pp. 19–28, 2024, doi: 10.24920/004338.

[11] N. Di Giorgi *et al.*, "Salivary Proteomics Markers for Preclinical Sjögren's Syndrome: A Pilot Study," *Biomolecules*, vol. 12, no. 6, Jun. 2022, doi: 10.3390/biom12060738.

[12] F. Finamore *et al.*, "Characterization of extracellular vesicle cargo in Sjögren's syndrome through a swath-ms proteomics approach," *Int J Mol Sci*, vol. 22, no. 9, May 2021, doi: 10.3390/ijms22094864.

[13] L. A. Aqrawi, J. L. Jensen, S. Fromreide, H. K. Galtung, and K. Skarstein, "Expression of NGAL-specific cells and mRNA levels correlate with inflammation in the salivary gland, and its overexpression in the saliva, of patients with primary Sjögren's syndrome," *Autoimmunity*, pp. 333–343, 2020, doi: 10.1080/08916934.2020.1795140.

[14] X. Chen *et al.*, "Elevated cytokine levels in tears and saliva of patients with primary Sjögren's syndrome correlate with clinical ocular and oral manifestations," *Sci Rep*, vol. 9, no. 1, Dec. 2019, doi: 10.1038/s41598-019-43714-5.

[15] A. Cecchettini *et al.*, "Phenotyping multiple subsets in Sjögren's syndrome: A salivary proteomic SWATH-MS approach towards precision medicine," *Clin Proteomics*, vol. 16, no. 1, Jun. 2019, doi: 10.1186/s12014-019-9245-1.

[16] L. A. Aqrawi *et al.*, "Proteomic and histopathological characterisation of sicca subjects and primary Sjögren's syndrome patients reveals promising tear, saliva and extracellular vesicle disease biomarkers," *Arthritis Res Ther*, vol. 21, no. 1, Jul. 2019, doi: 10.1186/s13075-019-1961-4.

[17] F. Garza-García, G. Delgado-García, M. Garza-Elizondo, L. Á. Ceceñas-Falcónc, D. Galarza-Delgado, and J. Riega-Torres, "Salivary B2-microglobulin positively correlates with ESSPRI in patients with primary Sjögren's syndrome," *Rev Bras Reumatol*, vol. 57, no. 2, pp. 182–184, 2017, doi: 10.1016/j.rbre.2016.11.001.

[18] L. A. Aqrawi *et al.*, "Identification of potential saliva and tear biomarkers in primary Sjögren's syndrome, utilising the extraction of extracellular vesicles and proteomics analysis," *Arthritis Res Ther*, vol. 19, no. 1, Jan. 2017, doi: 10.1186/s13075-017-1228-x.

[19] N. Delaleu, P. Mydel, J. G. Brun, M. V. Jonsson, A. Alimonti, and R. Jonsson, "Sjögren's syndrome patients with ectopic germinal centers present with a distinct salivary proteome," *Rheumatology (United Kingdom)*, vol. 55, no. 6, pp. 1127–1137, Jun. 2016, doi: 10.1093/rheumatology/kew013.

[20] O. Deutsch *et al.*, "Identification of Sjögren's syndrome oral fluid biomarker candidates following high-abundance protein depletion," *Rheumatology (United Kingdom)*, vol. 54, no. 5, pp. 884–890, Apr. 2014, doi: 10.1093/rheumatology/keu405.

[21] T. Cross *et al.*, "Non-Coding RNA in Salivary Extracellular Vesicles: A New Frontier in Sjögren's Syndrome Diagnostics?," *Int J Mol Sci*, vol. 24, no. 17, Sep. 2023, doi: 10.3390/ijms241713409.

[22] P. Karagianni, A. V. Goules, and A. G. Tzioufas, "Epigenetic alterations in Sjögren's syndrome patient saliva," *Clin Exp Immunol*, vol. 202, no. 2, pp. 137–143, Nov. 2020, doi: 10.1111/cei.13492.

[23] M. L. Sembler-Møller, D. Belstrøm, H. Locht, and A. M. L. Pedersen, "Distinct microRNA expression profiles in saliva and salivary gland tissue differentiate patients with primary Sjögren's syndrome from non-Sjögren's sicca patients," *Journal of Oral Pathology and Medicine*, vol. 49, no. 10, pp. 1044–1052, Nov. 2020, doi: 10.1111/jop.13099.

[24] S. Chiang *et al.*, "Distinctive profile of monomeric and polymeric anti-SSA/Ro52 immunoglobulin A1 isoforms in saliva of patients with primary Sjögren's syndrome and Sicca," *RMD Open*, vol. 10, no. 2, Apr. 2024, doi: 10.1136/rmdopen-2023-003666.

[25] P. Li, Y. Jin, R. Zhao, Z. Xue, and J. Ji, "Expression of ICOS in the salivary glands of patients with primary Sjogren's syndrome and its molecular mechanism," *Mol Med Rep*, vol. 26, no. 5, Nov. 2022, doi: 10.3892/mmr.2022.12864.

[26] L. A. Moreno-Quispe *et al.*, "Association of salivary inflammatory biomarkers with primary Sjögren's syndrome," *Journal of Oral Pathology and Medicine*, vol. 49, no. 9, pp. 940–947, Oct. 2020, doi: 10.1111/jop.13070.

[27] Y. Jin *et al.*, "Tissue-Specific Autoantibodies Improve Diagnosis of Primary Sjögren's Syndrome in the Early Stage and Indicate Localized Salivary Injury," *J Immunol Res*, vol. 2019, 2019, doi: 10.1155/2019/3642937.

[28] J. Lee *et al.*, "Soluble siglec-5 is a novel salivary biomarker for primary Sjogren's syndrome," *J Autoimmun*, vol. 100, pp. 114–119, Jun. 2019, doi: 10.1016/j.jaut.2019.03.008.

[29] P. Sandhya *et al.*, "Diagnostic accuracy of salivary and serum-free light chain assays in primary Sjögren's syndrome: a pilot study," *Int J Rheum Dis*, vol. 20, no. 6, pp. 760–766, Jun. 2017, doi: 10.1111/1756-185X.12965.

[30] L. Garreto *et al.*, "Mapping Salivary Proteases in Sjögren's Syndrome Patients Reveals Overexpression of Dipeptidyl Peptidase-4/CD26," *Front Immunol*, vol. 12, Jun. 2021, doi: 10.3389/fimmu.2021.686480.

[31] P. Wei, Y. Xing, B. Li, F. Chen, and H. Hua, "Proteomics-Based Analysis Indicating $\alpha$-Enolase as a Potential Biomarker in Primary Sjögren's Syndrome," *Gland Surg*, vol. 9, no. 6, pp. 2054–2063, Dec. 2020, doi: 10.21037/GS-20-814.
